# Supplementary material for: Primary and secondary clarithromycin resistance in Helicobacter pylori and mathematical modeling of the role of macrolides
Source: Nat Commun. 2021 Apr 15;12:2255. doi: 10.1038/s41467-021-22557-7 (PMC8050269; doi:10.1038/s41467-021-22557-7)
Supplement: Supplementary file 1 — Supplementary Information [file 41467_2021_22557_MOESM1_ESM.pdf]

Supplementary Information

Primary and secondary clarithromycin resistance in *Helicobacter pylori* and mathematical modeling of the role of macrolides

Éva Kocsmár[1], György Miklós Buzás[2], Ildikó Szirtes[1], Ildikó Kocsmár[1], Zsófia Kramer[1], Attila Szijártó[3], Petra Fadgyas-Freyler[4], Kató Szénás[5], Massimo Rugge[6,7], Matteo Fassan[6], András Kiss[1], Zsuzsa Schaff[1], Gergely Röst[8,9], Gábor Lotz[1]*

[1]2nd Department of Pathology, Semmelweis University, Budapest, Hungary
[2]Department of Gastroenterology, Ferencváros Health Center, Budapest, Hungary
[3]1st Department of Surgery and Interventional Gastroenterology, Semmelweis University, Budapest, Hungary
[4]National Health Insurance Fund Administration, Budapest, Hungary
[5]Department of Pathology, Péterfy Hospital, Budapest, Hungary
[6]Department of Medicine (DIMED), Surgical Pathology & Cytopathology Unit, University of Padua, Padua, Italy
[7]Veneto Tumor Registry (RTV), Veneto Regional Authority, Padua, Italy
[8]Wolfson Center for Mathematical Biology, University of Oxford, Oxford, United Kingdom
[9]Bolyai Institute, University of Szeged, Szeged, Hungary

* Correspondence to: lotz.gabor@med.semmelweis-univ.hu

Supplementary Note 1. Parameters of the epidemiological model.

Development of heteroresistance due to macrolide use (η: row 2 in Table 3 of the main text)

Data on the prior macrolide use of the patients of our cohort were obtained from the National Health Insurance Fund Management of Hungary. Their data protection/privacy policy allows them to provide only aggregated data for research purposes. Therefore, they collected information from health insurance databases and analyzed individual patients' prior macrolide use. Then, on this basis, they provided us with data on macrolide use in the groups/subgroups for which it was previously requested. As a practical consideration, since the study was primarily designed to identify macrolide-naive (primary resistant) infections, "any prior macrolide use" was investigated during the data collection. Thus, identifying the homo- and heteroresistant cases separately in each subgroup of clarithromycin-resistant (Cla-res) infections was not included in our primary goals; therefore, unfortunately, the macrolide-use-related homo-/heteroresistance ratio is not available in detail from each aspect in this cohort, including non-eradication-related clarithromycin / other macrolide use. However, we can calculate this "Development of heteroresistance due to macrolide use" parameter (corresponding to non-eradication-related macrolide use) indirectly by considering the data from one of our previous publications.[1]

In that published study, we investigated the homo-/heteroresistance ratio among patients who acquired secondary resistance due to unsuccessful clarithromycin-based eradication attempts.[1] We found a 70%/30% homo-/heteroresistance ratio (23 homoresistant and 10 heteroresistant cases) among all cases of clarithromycin resistance related to unsuccessful eradication attempts.

In the present cohort, we observed 247 Cla-res cases with a history of previous clarithromycin-based eradication attempts (see Table 2 and the main text) and 297 macrolide-exposed heteroresistant cases (secondary heteroresistance, Supplementary Table 1). If we apply the 70%/30% ratio to the 247 patients with unsuccessful eradication treatment, then it will result in 173 homoresistant and 74 heteroresistant cases. These 74 unsuccessfully eradicated heteroresistant cases were subtracted from the 297 heteroresistant patients exposed to any macrolide, resulting in 223 heteroresistant cases out of the 429 individuals in the non-eradication-related macrolide-exposed group. On this basis, the homo-/heteroresistance ratio among these patients was 48%/52% (206 homoresistant and 223 heteroresistant). Considering that the 429 resistant patients accounted for 24.8% of the total 1731 non-eradication-related macrolide-exposed persons, the "Development of heteroresistance due to macrolide use" parameter was set to 12.9% (0.129) based on the 48%/52% homo-/heteroresistance ratio.

	Total	Females	Males	p	CI of OR	CI of RR	p	CI of OR	CI of RR	p (2x3)*
	4744	2709	2035	In total						
**Macrolide-naive**	2532	1313	1219	**6.164 × 10^{-15}**	1.41-1.78	1.16-1.28	In macrolide-naive subjects			
Macrolide-naive susceptible	2392	1229	1163	**1.072 × 10^{-15}**	1.43-1.80	1.17-1.29	0.0553	0.99-2.05	1.02-1.34	
Primary homoresistance	52	30	22	1.0000	0.56-1.69	0.78-1.25	0.4046	0.43-1.42	0.71-1.14	0.1282
Primary heteroresistance	88	54	34	0.4478	0.54-1.29	0.79-1.10	0.0819	0.42-1.06	0.71-1.00	
Total primary resistance	140	84	56	0.5442	0.63–1.25	0.83–1.10	0.0553	0.49-1.01	0.74-0.99	
**Macrolide-exposed**	2212	1396	816	**6.164 × 10^{-15}**	0.56-0.71	0.78-0.86	In macrolide-exposed subjects			
Macrolide-exposed susceptible	1536	943	593	**3.508 × 10^{-5}**	0.68-0.87	0.85-0.94	**0.0128**	1.05-1.55	1.02-1.17	
Secondary homoresistance	379	259	120	**3.905 × 10^{-6}**	0.47-0.75	0.76-0.88	**0.0225**	0.59-0.96	0.84-0.98	**0.0297**
Secondary heteroresistance	297	194	103	**0.003**	0.54-0.89	0.79-0.94	0.4381	0.69-1.16	0.88-1.05	
Total secondary resistance	676	453	223	**1.71 × 10^{-8}**	0.51-0.73	0.78-0.88	**0.0128**	0.65-0.95	0.86-0.98	

Supplementary Table 1

Distribution of clarithromycin-susceptible, homoresistant and heteroresistant cases in the macrolide-naive and macrolide-exposed groups of *H. pylori*-infected patients. The macrolide-exposed group included all patients with any type of prior macrolide intake (eradication-related clarithromycin, non-eradication-related clarithromycin, other macrolide use). The p values of the female-male comparisons were calculated by a two-tailed Fisher's exact test. Significant results are highlighted in bold. *2×3 two-tailed Fisher's exact test for female-male comparison of the homoresistant, heteroresistant and susceptible cases in the primary (macrolide-naive) and secondary (macrolide-exposed) clarithromycin-resistant groups of *H. pylori* infections.

Successful eradication (ρ_u: row 3 in Table 3 of the main text)

This parameter (the rate of successful *H. pylori* eradication with a clarithromycin-containing regimen) corresponds to the success rate of clarithromycin-based eradication therapy in Cla-susceptible infections. To set this parameter, we read studies with a susceptibility-guided approach in which Cla-susceptible cases were treated by classic triple therapy, and an intention-to-treat analysis was performed.[2,3] Based on these published data, the parameter was set to 0.92, meaning that 92% of Cla-susceptible infections can be eradicated by clarithromycin-containing regimens.

No effect of Cla treatment (ρ_w: row 4 in Table 3 of the main text)

This parameter corresponds to the rate of cases in which the clarithromycin-containing eradication treatment failed, the host remained infected and the remaining *H. pylori* population was Cla-susceptible. This can be calculated from the previous parameter ("Successful eradication"). Since the rate of successful *H. pylori* eradication with clarithromycin-containing treatment is 92%, the remaining 8% corresponds to the 481 patients who had previously had unsuccessful eradication attempts (main text Table 2, first section, "Cla eradication attempt"/"Total"). This group is composed of 234 Cla-susceptible cases (corresponding to the "No effect of Cla treatment" parameter) as well as 247 Cla-res cases, which can be further divided into homo- and heteroresistant subgroups (Cla-treatment-induced homoresistance/heteroresistance; see below). As the 8% were divided in a ratio of 234:247, the "No effect of Cla treatment" parameter was set to 3.89% (0.0389).

Cla-treatment-induced homoresistance/Cla-treatment-induced heteroresistance (ρ_r and ρ_h: rows 5-6 in Table 3 of the main text)

These parameters correspond to the rates of cases in which the clarithromycin-containing eradication treatment failed, the host remained infected and the remaining *H. pylori* population was either homoresistant or heteroresistant to clarithromycin. As we have discussed above in connection with the parameter "Development of heteroresistance due to macrolide use", we have investigated the homoresistance/heteroresistance ratio among patients who acquired secondary resistance by unsuccessful clarithromycin-based eradication attempts in one of our previous studies.[1] We found a 70%/30% homoresistance/heteroresistance ratio (23 homoresistant and 10 heteroresistant cases) among the unsuccessful-eradication-induced Cla-res cases. Moreover, as we have deduced above, the group comprising the 8% of previously unsuccessfully eradicated patients was divided in a ratio of 234:247 (no effect of Cla treatment/resistance induced by Cla-containing treatment); consequently, Cla-induced resistance (247 cases) corresponded to 4.11%. We divided this group further in a ratio of 23:10, resulting in 2.86% (0.0286) Cla-treatment-induced homoresistance and 1.25% (0.0125) heteroresistance.

Cla treatment rate

(θ: row 7 in Table 3 of the main text)

This parameter corresponds to the annual rate of cases undergoing Cla-containing *H. pylori* eradication treatment in the population.
Although we determined the rate of previous clarithromycin-containing eradication treatment in our cohort, we did not accept this rate as representative of the whole population of Central Hungary. Specifically, these *H. pylori*-infected gastroenterology patients represent a special subset of the population displaying the highest rate of previous eradication attempts with clarithromycin-containing regimens. Therefore, this might correspond to the maximum clarithromycin-based *H. pylori* eradication treatment rate in the whole population of Central Hungary. Since the age distributions of our cohort and the Central Hungarian population were different, the rates of clarithromycin-containing eradication treatment were determined separately for each female and male age group in our cohort. These rates were used to calculate the number of affected individuals in the same age group within the Central Hungarian population. Finally, these numbers were added together (see the final row (”Total”) of Supplementary Table 2, in the column “Population Eradicated”).
However, we decided to let θ vary freely instead of setting an upper limit.

Rate of macrolide consumption

(m: row 8 in Table 3 of the main text)

This parameter corresponds to the annual rate of cases undergoing macrolide treatment (with clarithromycin or other macrolide antibiotics) for purposes other than eradication.
To assess this annual rate, we aimed first to evaluate the rate of macrolide treatment (with clarithromycin or any other macrolide) whose purpose was not eradication. We have accepted the non-eradication-related macrolide use rates observed in this cohort as representative of the whole Central Hungarian population. Specifically, the *H. pylori*-related health problems of our patients do not predispose them to use more macrolides for non-eradication purposes than other individuals in the population, since the other common indications for these antibiotics include the treatment of upper and lower respiratory tract infections, pelvic inflammatory disease, and skin and soft tissue infections but not gastrointestinal diseases. Since the age distribution of our cohort was different from that of the Central Hungarian population, the rates of non-eradication-related macrolide consumption were determined separately for each female and male age group in our cohort. These rates were used to calculate the number of affected individuals in the same age group within the Central Hungarian population. Finally, these numbers were added together (see the final row (”Total”) of Supplementary Table 2, in the column “Population: Macrolide exposed”), and the proportion relative to the whole population was accepted as the total rate of non-eradication-related macrolide consumption in the population of Central Hungary. Considering the assessed 40.06% rate of prior non-

eradication-purpose macrolide use in the population of the Central Hungary Region, the annual rate was calculated to be $m = 0.0087$ (for further details, see Supplementary Note 2).

Gender	Age groups *(years)*	Cohort: Total *(n)*	Cohort: Macrolide exposed *(n, %)*	Cohort: Eradicated *(n, %)*	Population: Total *(n)*	Population: Macrolide exposed *(n, %)*	Population: Eradicated *(n, %)*
Male	0-19	18	10 (55.56%)	2 (11.11%)	292,440	*162,467.67 (55.56%)*	*32,493.33 (11.11%)*
	20-29	116	46 (39.66%)	1 (0.86%)	191,642	*75,995.97 (39.66%)*	*1,652.09 (0.86%)*
	30-39	256	81 (31.64%)	13 (5.08%)	257,633	*81,516.69 (31.64%)*	*13,082.93 (5.08%)*
	40-49	311	76 (24.44%)	33 (10.61%)	188,944	*46,172.81 (24.44%)*	*20,048.72 (10.61%)*
	50-59	513	149 (29.04%)	50 (9.75%)	177,840	*51,653.33 (29.04%)*	*17,333.33 (9.75%)*
	60-69	444	153 (34.46%)	38 (8.56%)	152,178	*52,439.72 (34.46%)*	*13,024.24 (8.56%)*
	70+	377	119 (31.56%)	45 (11.94%)	117,358	*37,044.04 (31.56%)*	*14,008.25 (11.94%)*
	Subtotal	2035	634 (31.16%)	182 (8.94%)	1,378,035	*507,289.22 (36.81%)*	*111,642.89 (8.10%)*
Female	0-19	34	17 (50.0%)	4 (11.75%)	278,190	*139,095.00 (50.0%)*	*32,728.24 (11.75%)*
	20-29	136	58 (42.65%)	4 (2.94%)	194,402	*82,906.74 (42.65%)*	*5,717.71 (2.94%)*
	30-39	280	132 (47.14%)	30 (10.71%)	264,389	*124,640.53 (47.14%)*	*28,327.39 (10.71%)*
	40-49	429	176 (41.03%)	54 (12.59%)	195,133	*80,054.56 (41.03%)*	*24,562.20 (12.59%)*
	50-59	686	300 (43.73%)	89 (12.97%)	210,924	*92,240.82 (43.73%)*	*27,364.78 (12.97%)*
	60-69	542	218 (40.22%)	65 (11.99%)	203,754	*81,952.72 (40.22%)*	*24,435.44 (11.99%)*
	70+	602	196 (32.56%)	53 (8.80%)	221,689	*72,177.81 (32.56%)*	*19,517.47 (8.80%)*
	Subtotal	2709	1097 (40.50%)	299 (11.04%)	1,568,481	*673,068.17 (42.91%)*	*162,653.22 (10.37%)*
Total		4744	1731 (36.49%)	481 (10.14%)	2,946,516	*1,180,357.39 (40.06%)*	*274,296.11 (9.31%)*

Supplementary Table 2 Calculation of population-level macrolide use and history of eradication with clarithromycin-containing regimens. Calculated values are shown in italic.

Spontaneous mutation rate (ω: row 9 in Table 3 of the main text)

According to Linz *et al.* A mutation burst during the acute phase of Helicobacter pylori infection in humans and rhesus macaques. *Nat Commun.* **5**, 4165 (2014).[4]

Calculation of the prevalence of *H. pylori* in Central Hungary
(H: row 13 in Table 3 of the main text)

The prevalence of *H. pylori* in the studied population was needed for mathematical modeling of the population dynamics of Cla-res *H. pylori* infection. However, no representative study addresses the prevalence of *H. pylori* in Central Hungary during the study period. A study from southeast Hungary investigated the prevalence of *H. pylori* in 1001 healthy blood donor volunteers and found it to be 32%.[5] However, we did not consider this study to be representative of the general population of Central Hungary. This decision was based mostly on the fact that this prior study included healthy volunteers only; consequently, the cohort of Balint et al. had a significantly lower mean age than our cohort (40 vs. 55 years). Thus, their prevalence data were found to be not representative of the whole population of Central Hungary, as age has a significant influence on *H. pylori* prevalence. Other reasons included the regional differences between the cohort of Balint et al. and the population of Central Hungary. Balint et al. investigated Csongrád and Békés Counties, which are situated in southeastern Hungary and have more agricultural workers and a lower population density than the capital city and Pest County. In contrast, Buzás et al.[6] collected prevalence data from the population of Central Hungary, and their prevalence data grouped according to birth cohort allowed us to extrapolate to the age-grouped population data of the Hungarian population census from the investigated years. We considered this solution to be the closest estimate of the real prevalence data from the Central Hungary Region.
Thus, to assess prevalence, we used *H. pylori* infection rates by birth cohort from a previous study by Buzás et al.[6] These rates were as follows:

Birth cohort	1920–29	1930–39	1940–49	1950–59	1960–69	1970–79	1980–89	1990–94
H. pylori prevalence, %	63.6	62.8	63.0	62.1	57.4	39.0	26.7	12.2

Birth cohort	1960–64	1965–69	1970–74	1975–79
H. pylori prevalence, %	61.4	53.9	44.5	34.2

Supplementary Table 3 *H. pylori* prevalence data from Buzás et al.[6] by birth cohort.

Then, these prevalence data were fitted to the birth cohorts of the age-grouped population data of the Hungarian population census 2011. This fitting was necessary because the birth cohorts from this population census and from the study of Buzás et al.[6] both have 10-year/5-year ranges (periods), but these were "shifted" relative to each other. This means that in the study of Buzás et al.[6], these birth cohorts were, for example, 1940-49, 1950-59, 1960-64, 1965-69, etc. while the population census used five-year periods such as 1952-56, 1957-1961, 1962-66, 1967-71, etc. Consequently, there are certain 5-year periods of the population census that completely overlap with one of the 10-year periods of study of Buzás et al.[6] (e.g., 1932-1936 with 1930-39). For these 5-year periods of the population census, we used the prevalence data from the related 10-year period in the study by Buzás et al.[6] However, regarding those 5-year population census periods that overlap with two periods of the study from Buzás et al.[6] (e.g., 1937-1941 with 1930-39 and 1940-49, or 1972-1976 with 1970-74 and 1975-79), we calculated a weighted average by obtaining the prevalence for 3 years of the 5-year period from the earlier decade and for 2 years of the 5-year period from the later decade in the study by Buzás et al.[6] (e.g., 1930-39: 62.8% (Buzás), 1940-49: 63.0% (Buzás); calculated prevalence for the 1937-1941 period: [62.8x0.6]+[63.0x0.4)=62.88%). The only exception was the 1992-1996 period, since we had no data for the final 2 years of that period; therefore, we applied the last available 1990-94 prevalence data (12.2%) to the whole period from 1992 to 1996.
However, no data were available for the 2007–2011, 2002–2006 or 1997–2001 birth cohort; therefore, we extrapolated these rates by fitting a linearly increasing trend with age: 1.7% (2007-2011), 5.2% (2002-2006) and 8.7% (1997-2001) (shown in italics in Supplementary Table 4).
Then, we calculated the number of infected persons separately for each birth cohort from its population size and the calculated or assumed prevalence data, as shown in the right-hand column of Supplementary Table 4. Finally, the population prevalence of *H. pylori* was calculated as the ratio of the total number of infected persons to the total population, resulting in a total prevalence of 40.9% (Supplementary Table 4). Therefore, this 40.9% was used as the *H. pylori* prevalence in the mathematical model of transmission dynamics of Cla-res *H. pylori*.

Data source (population census 2011, Hungary):
Budapest
http://www.ksh.hu/nepszamlalas/docs/tables/regional/01/01_1_1_2_1_en.xls
Pest County
http://www.ksh.hu/nepszamlalas/docs/tables/regional/13/13_1_1_2_1_en.xls

Age group, years	Population census 2011, Hungary			Birth cohort	*H. pylori* prevalence (%)	Number of infected persons
	Population					
	Budapest	Pest County	Total			
–4	80,067	68,754	148,821	2007–2011	*1.7*	2530
5–9	68,658	69,504	138,162	2002–2006	*5.2*	7184
10–14	61,915	66,011	127,926	1997–2001	*8.7*	11,130
15–19	81,626	74,095	155,721	1992–1996	12.2	18,998
20–24	115,949	71,187	187,136	1987–1991	20.9	39,111
25–29	130,178	68,730	198,908	1982–1986	26.7	53,108
30–34	160,617	96,307	256,924	1977–1981	31.2	80,160
35–39	152,923	112,175	265,098	1972–1976	40.4	107,047
40–44	121,517	97,422	218,939	1967–1971	50.1	109,776
45–49	91,850	73,288	165,138	1962–1966	58.4	96,441
50–54	93,377	73,570	166,947	1957–1961	61.8	103,207
55–59	132,002	89,815	221,817	1952–1956	62.1	137,748
60–64	114,020	77,446	191,466	1947–1951	62.6	119,934
65–69	102,808	61,658	164,466	1942–1946	63.0	103,614
70–74	75,876	45,495	121,371	1937–1941	62.9	76,318
75–79	59,715	33,232	92,947	1932–1936	62.8	58,371
80–84	47,728	22,643	70,371	1927–1931	63.3	44,531
85+	38,214	16,144	54,358	Before 1927	63.6	34,572
Total	1,729,040	1,217,476	2,946,516			1,203,779
					Total *H. pylori* prevalence:	40.9%

Supplementary Table 4

Calculation of *H. pylori* prevalence in the Central Hungarian population

Supplementary Note 2: The epidemiological model.

1 Model description

To estimate some key parameters, we construct a mathematical model for the transmission dynamics of *H. pylori*. We stratify a population into compartments according to infection status (uninfected, infected with wild-type strain only, infected with resistant strain only (homoresistant), heteroresistant infection) and medication history (used clarithromycin for eradication purposes, used macrolides for other purposes, macrolide naive). This produces twelve compartments altogether, which are given in Supplementary Table 5. The notation is straightforward; for example $i_r^c(t)$ denotes the fraction of the population that has history of eradication purpose use of clarythromicin, and is infected with a resistrant strain at time t.

	uninfected	wild-type infected	homoresistant	heteroresistant
macrolide naive	u^n	i_w^n	i_r^n	i_h^n
macrolide history	u^m	i_w^m	i_r^m	i_h^m
clarithromycin history	u^c	i_w^c	i_r^c	i_h^c

Supplementary Table 5: Model compartments.

The transitions between compartments are as follows. The population size is normalized to unity, and assumed to be in demographic balance by setting the birth and death rates equal (μ), where newborns appear in the macrolide naive uninfected compartment. We consider three types of resistance status: wild-type (index w; with clarithromycin susceptible bacteria), homoresistant (index r; with bacteria resistant to clarithromycin) and heteroresistant (index h; with simultaneous presence of both wild-type and resistant bacteria). Uninfected individuals can acquire the infection from wild or resistant type infected hosts, then they move to the corresponding infected compartment. Heteroresistant hosts expose infectees with wild-type bacteria with probability κ and resistant type with probability $1 - \kappa$ (thus κ can be interpreted as relative bacterial shedding of the wild-type by heteroresistant hosts), and transmission occurs with rate β and $\delta\beta$; where the factor δ expresses the relative transmission fitness of resistant strains. We assume that infection with either wild-type or resistant strain does not confer protection against the other strain, meaning that hosts infected with the wild-type strain can be overcolonized by the homoresistant and vice versa, the homoresistant infections by the wild-type strains with the same transmission rates, upon which they become heteroresistant. Individuals in the macrolide naive compartments are using macrolides with rate m, and move to the corresponding compartment with

history of macrolide use, but in a fraction η of wild-type infecteds resistance emerges and they become heteroresistant. Infected individuals with or without a history of macrolide use undergo clarithromycin treatment for the purpuse of eradication with a rate of θ. Such treatment can have various outcomes: in case of a wild-type infected, the treatment can be successful and lead to eradication (with probability ρ_u) and transition back to the uninfected compartment; the treatment can fail such that the hosts remain wild-type infected (with probability ρ_w); or the resistance may emerge in the hosts placing them into resistant or heteroresistant compartments (with probabilities ρ_r and ρ_h), where $\rho_u + \rho_w + \rho_r + \rho_h = 1$. If clarithromycin treatment is administered to a heteroresistant host, it may result a homoresistant infection by eradication of the wild-type strain (with probability $\rho_u + \rho_r$, respectively), or the host remains heteroresistant (with probability $\rho_h + \rho_w$). Treatment has no effect on homoresistant infected hosts. We allow the possibility of emergence of resistance due to spontaneous mutations, expressed by a transition with rate ω from a wild-type infected compartment to the corresponding heteroresistant compartment. The transmission diagram is depicted in Supplmentary Fig. 1.

2 The governing equations

Based on the model description above, the governing equations are written as

$$\begin{aligned}
\frac{du^n(t)}{dt} &= \mu - (\mu + m)u^n(t) - (\lambda_w(t) + \lambda_r(t))u^n(t), \\
\frac{di^n_w(t)}{dt} &= \lambda_w(t)u^n(t) - (\mu + m)i^n_w(t) - \lambda_r(t)i^n_w(t) - \theta i^n_w(t) - \omega i^n_w(t), \\
\frac{di^n_r(t)}{dt} &= \lambda_r(t)u^n(t) - (\mu + m)i^n_r(t) - \lambda_w(t)i^n_r(t) - \theta i^n_r(t), \\
\frac{di^n_h(t)}{dt} &= \lambda_r(t)i^n_w(t) + \lambda_w(t)i^n_r(t) - (\mu + m)i^n_h(t) - \theta i^n_h(t) + \omega i^n_w(t),
\end{aligned}$$

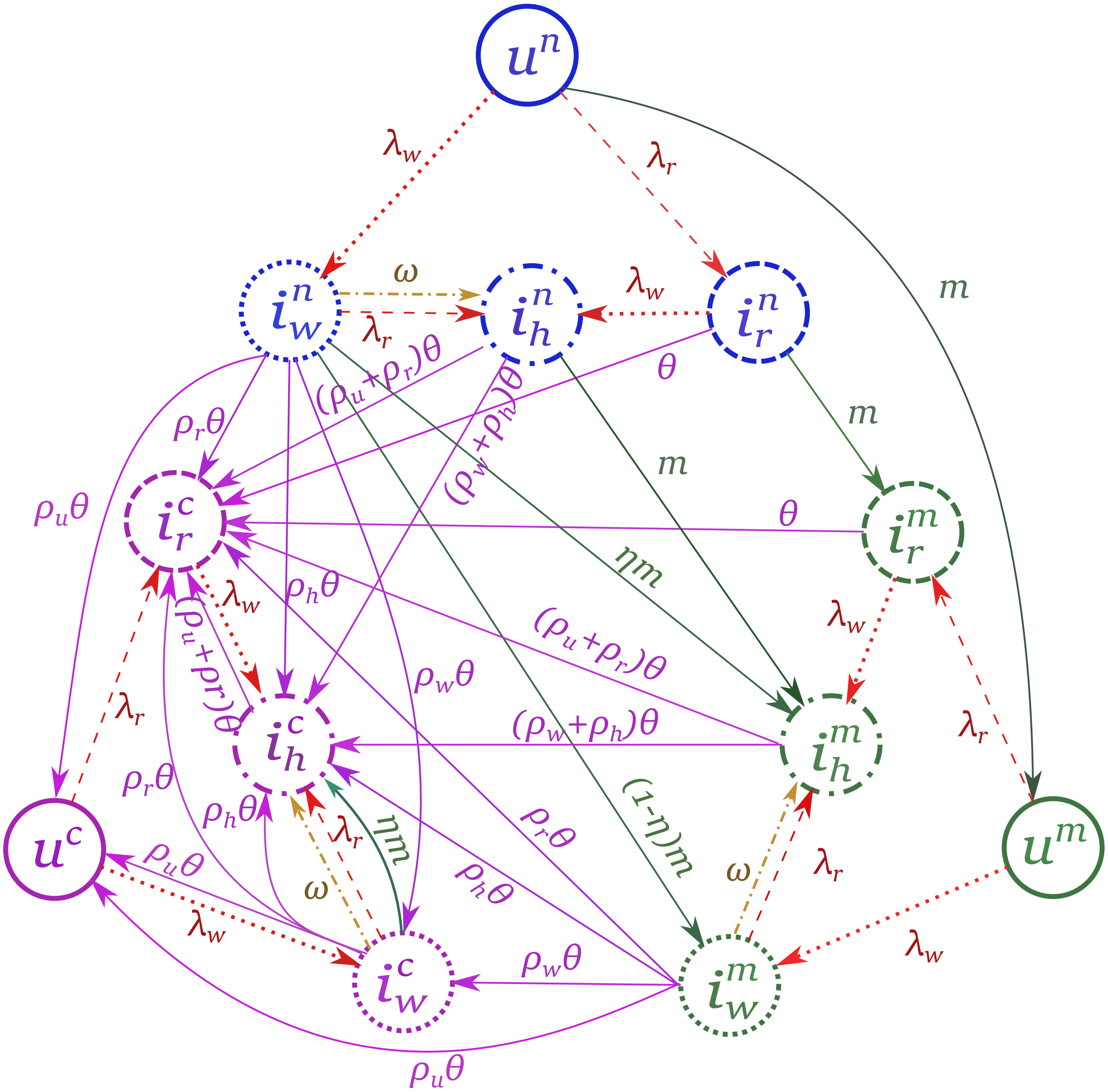

Legend:

infection with wild type
infection with resistant type

spontaneous mutation

Cla-eradication attempt
other macrolide use

Supplementary Fig. 1: Model transfer diagram.

$$
\begin{aligned}
\frac{du^m(t)}{dt} &= -\mu u^m(t) - (\lambda_w(t) + \lambda_r(t))u^m(t) + mu^n(t), \\
\frac{di_w^m(t)}{dt} &= \lambda_w(t)u^m(t) - \mu i_w^m(t) - \lambda_r(t)i_w^m(t) - \theta i_w^m(t) - \omega i_w^m(t) \\
&\quad +(1-\eta)mi_w^n(t), \\
\frac{di_r^m(t)}{dt} &= \lambda_r(t)u^m(t) - \mu i_r^m(t) - \lambda_w(t)i_r^m(t) - \theta i_r^m(t) + mi_r^n(t), \\
\frac{di_h^m(t)}{dt} &= \lambda_r(t)i_w^m(t) + \lambda_w(t)i_r^m(t) - \mu i_h^m(t) - \theta i_h^m(t) + mi_h^n(t) + \eta mi_w^n(t) \\
&\quad +\omega i_w^m(t),
\end{aligned}
$$

$$
\begin{aligned}
\frac{du^c(t)}{dt} &= -\mu u^c(t) - (\lambda_w(t) + \lambda_r(t))u^c(t) + \rho_u\theta(i_w^n(t) + i_w^m(t) + i_w^c(t)), \\
\frac{di_w^c(t)}{dt} &= \lambda_w(t)u^c(t) - \mu i_w^c(t) - \lambda_r(t)i_w^c(t) - \omega i_w^c(t) - \theta i_w^c(t) - m\eta i_w^c(t) \\
&\quad +\rho_w\theta(i_w^n(t) + i_w^m(t) + i_w^c(t)), \\
\frac{di_r^c(t)}{dt} &= \lambda_r(t)u^c(t) - \mu i_r^c(t) - \lambda_w(t)i_r^c(t) - \theta i_r^c(t) + \rho_r\theta(i_w^n(t) + i_w^m(t) + i_w^c(t)) \\
&\quad +\theta(i_r^n(t) + i_r^m(t) + i_r^c(t)) + (\rho_u + \rho_r)\theta(i_h^n(t) + i_h^m(t) + i_h^c(t)), \\
\frac{di_h^c(t)}{dt} &= \lambda_r(t)i_w^c(t) + \lambda_w(t)i_r^c(t) - \mu i_h^c(t) - \theta i_h^c(t) + \omega i_w^c(t) + m\eta i_w^c(t) \\
&\quad +\rho_h\theta(i_w^n(t) + i_w^m(t) + i_w^c(t)) + (\rho_w + \rho_h)\theta(i_h^n(t) + i_h^m(t) + i_h^c(t)),
\end{aligned}
$$

where the force of infections for wild-type and resistant types are given by

$$
\begin{aligned}
\lambda_w(t) : &= \beta(i_w^n(t) + i_w^m(t) + i_w^c(t) + \kappa(i_h^n(t) + i_h^m(t) + i_h^c(t))), \\
\lambda_r(t) : &= \delta\beta(i_r^n(t) + i_r^m(t) + i_r^c(t) + (1-\kappa)(i_h^n(t) + i_h^m(t) + i_h^c(t))).
\end{aligned}
$$

3 Limitations

Our epidemiological model accounts for the most important state transitions due to infection, medication use and emergence of resistance. To keep the equations tractable, we made several simplifications. We categorized the bacterial clarithromycin resistance status into either wild-type or resistant type, and assumed that all bacteria within such categories share the same parameters. We ignored the effect of repeated macrolide use: while it may happen that the first macrolide use has no effect in a patient but a future macrolide use in the same patient induces resistance, such events are rare and

we assume it does not have significant impact on the overall epidemiological dynamics. We assumed that a heteroresistant infected host transmits either the wild-type (clarithromycin-susceptible) isolate/strain or the resistant isolate/strain during an infection episode and cannot transmit both wild-type and resistant bacteria at once to the same host. We assumed that spontaneous mutation and macrolide use in a host infected with wild-type (clarithromycin-susceptible) *H. pylori* give rise to heteroresistance, and that the emerged resistant strain cannot completely eliminate the already established wild-type population from within the host. A further assumption during the calculations was that the population is in demographic balance, and changing slowly epidemiologically. The model also has the usual limitations of compartmental models, such as it assumes a homogeneous, well-mixed population and mass-action incidence.

4 Calculations

4.1 Current prevalence and its trend

It is useful to introduce the quantity H to denote the prevalence of *H. pylori* in the population, that is

$$H := i_w^n + i_r^n + i_h^n + i_w^c + i_r^c + i_h^c + i_w^m + i_r^m + i_h^m.$$

Based on the detailed calculation of the prevalence in Supplementary Note 1, we set $H = 0.409$. It has been observed that the prevalence is slowly decreasing in several countries[7], in particular a 9% drop was observed in a 46 years period, which is a $\sim 0.2\%$ decrease by a year. Hence here we use $\frac{dH}{dt} = 0.002$ as a baseline, and assume a range of

$$-0.003 \leq \frac{dH}{dt} \leq -0.001,$$

where, using $u^n + u^m + u^c = 1 - H$,

$$\frac{dH}{dt} = (\lambda_r + \lambda_w)(1 - H) - \mu H - \rho_u \theta (i_w^n + i_w^m + i_w^c).$$

To estimate θ, later we shall fix the value of $\frac{dH}{dt}$ and use this relation in the form

$$\theta = \frac{(\lambda_r + \lambda_w)(1 - H) - \mu H - \frac{dH}{dt}}{\rho_u (i_w^n + i_w^m + i_w^c)}.$$

4.2 Current state variables

Based on the data from Tables 1–2 in the main text, and assuming that they are representative for a population, we infer the relations

$$i_w^n = (2392/4744) \times H,$$
$$i_w^c = (234/4744) \times H,$$
$$i_w^m = (1302/4744) \times H.$$

From Table 1 we can find

$$i_r^n + i_r^c + i_r^m = (431/4744) \times H,$$

$$i_h^n + i_h^c + i_h^m = (385/4744) \times H,$$

and from Table 2,

$$i_r^n + i_h^n = (140/4744) \times H,$$
$$i_r^m + i_h^m = (429/4744) \times H,$$
$$i_r^c + i_h^c = (247/4744) \times H.$$

From Supplementary Note 1, Supplementary Table 1 shows that

$$i_r^n = (52/4744) \times H,$$

and

$$i_h^n = (88/4744) \times H,$$

therefore we also find

$$i_r^c + i_r^m = (379/4744) \times H,$$
$$i_h^c + i_h^m = (297/4744) \times H.$$

4.3 Demographic turnover, macrolide consumption, and development of heteroresistance due to macrolide use or spontaneously

We assume that the population under study is in demographic balance, and we use the average mortality rate from the Hungarian Central Statistic Office[8] between 2005-2013, that is $\mu = 0.013$.

To obtain the macrolide consumption rate m, we use the auxiliary model

$$\frac{dx}{dt} = \mu - (\mu + m)x(t),$$

$$\frac{dy}{dt} = mx(t) - \mu y(t),$$

where x is the macrolide naive population, and y is the population with history of macrolide usage. The asymptotically stable steady state is $\bar{x} = \frac{\mu}{\mu+m}$, $\bar{y} = \frac{m\bar{x}}{\mu} = \frac{m}{\mu+m}$, hence $m = \frac{\mu\bar{y}}{1-\bar{y}}$. From the data of Supplementary Note 1, we find $\bar{y} = 0.406$, hence $m = 0.00889$.

From Supplementary Note 1, Table 2, one can see that 42.91% of females, and 36.81% of males has a history of macrolide use. For the sake of comparison, we calculate the macrolide consumption rates separately for females (m_{female}) and males (m_{male}) as well. The same calculation as for the total population above gives $m_{female} = 0.00977$ and $m_{male} = 0.00757$.

Also in Supplementary Note 2, $\eta = 0.129$ has been calculated, and here we use this value and the range $[0.12, 0.14]$ for the probability η of developing resistance via macrolide consumption.

For the spontaneous mutation rate we use $\omega = 1.55 * 10^{-5}$, as explained in Supplementary Note 1.

4.4 Treatment parameters

We use the arguments of Supplementary Note 1 to set our parameters related to treatment outcome. For successful eradication, we set $\rho_u = 0.92$, with the range $(0.88, 0.96)$. The remaining fraction 0.08 was divided into $\rho_w = 0.0389$ (treatment had no effect whatsoever), $\rho_r = 0.0286$ (treatment leads to homoresistance) and $\rho_h = 0.0125$ (treatment leads to heteroresistance). Note that if we use $\rho_u = 0.96$ in the sensitivity analysis, then the values of ρ_h, ρ_r, ρ_w will be decreased to their half (and similarly, increased by its half for $\rho_u = 0.88$), since the proportions among these parameters remain the same (see the argument in Supplementary Note 1).

4.5 Parameters and current states

The model parameters are summarized in Supplementary Table 6. The remaining parameters and values of state variables are inferred from the compartmental model as outlined below.

notation	parameter	value/range (reference)
μ	demographic turnover	0.013
η	development of heteroresistance due to macrolide use	0.129 (0.12 - 0.14)
ρ_u	successful eradication	0.92 (0.88 - 0.96)
ρ_w	no effect of Cla-treatment	0.0389
ρ_r	Cla-treatment induced homoresistance	0.0286
ρ_h	Cla-treatment induced heteroresistance	0.0125
θ	Cla-treatment rate	to be estimated, > 0
m	rate of macrolide consumption	0.00889
ω	spontaneous mutation rate	0.0000155
β	transmission rate	to be estimated, > 0
δ	relative transmission parameter	to be estimated, > 0
κ	resistant/wild-type exposure ratio for heteroresistants	$0 - 1$
H	prevalence of *H. pylori*	0.409
$\frac{dH}{dt}$	prevalence trend	-0.002 (-0.003 – -0.001)

Supplementary Table 6: A-priori model parameters

Consider our system in the form $x' = F(x)$, where $F_i(x, p)$ is the given component of the right hand side of the model equations, with variables x and parameters p. We assume that the epidemiological dynamics is slow, with the constraint that the prevalence is decreasing by a prescribed rate. Let $W(x, p) = \sum_{i=1}^{12} \left(\frac{F_i(x,p)}{x_i}\right)^2$, the sum of squares of relative variations of the state variables. We minimize W over the domain of feasible parameters and constraints. The obtained numerical values are given in Supplementary Table 7. The calculations were performed with Wolfram Mathematica software, see Supplementary Note 3 for the code and the details.

5 Sensitivity analysis

We have incorporated as much a-priori information about the parameters and the state variables as possible. Given the uncertainty in some parameters, we have performed a sensitivity analysis with respect to three quantities, and explored how the outcome changes as we sweeping through the cube $[-0.003, -0.001] \times [0.88, 0.96] \times [0.12, 0.14] \ni$

μ	0.013	i_r^n	0.00448314
η	0.129	i_r^m	*0.0185289*
ρ_u	0.92	i_r^c	*0.0141463*
ρ_w	0.039	i_w^n	0.206224
ρ_r	0.029	i_w^m	0.112251
ρ_h	0.012	i_w^c	0.0201741
θ	*0.00295584*	i_h^n	0.00758685
m	*0.00889*	i_h^m	*0.018457*
ω	0.0000155	i_h^c	*0.00714859*
λ_r	*0.00040911*	u_n	*0.390557*
λ_w	*0.00676164*	u_m	*0.154876*
H	0.409	u_c	*0.0455673*

Supplementary Table 7: Parameters and current states. Those with italic are inferred from the numerical model calculations, the rest are estimated from data and literature.

$(\frac{dH}{dt}, \rho_u, \eta)$, and the ranges around the main results are coming from this sensitivity analysis. We have found that varying ρ_u and η has minuscule effect on the outcome (at least in this range), and almost all the variation is generated by varying $\frac{dH}{dt}$.

6 Conclusions

From the numbers above we can draw the following conclusions. The proportion of new infections in the population that arise from the transmission of the resistant strain is $\lambda_r/(\lambda_r + \lambda_w)$, which yields approximately 5.7% (4.67% − 7.43%). From this, assuming $\kappa = 0.5$, that is a heteroresistant infected exposes the infectee to both strains with equal probability, we obtain that the relative transmission fitness of the resistant strain is $\delta = 0.72$ $(0.58 - 0.95)$, and the transmission rate is $\beta = 0.019$ $(0.014 - 0.024)$. Letting κ vary in the interval $[0, 1]$, in the baseline case the possible range of δ is $[0.5, 1.45]$, and the possible range of β is $[0.018, 0.02]$.

The origin of the resistant type infection in the macrolide naive subpopulation $(i_h^n + i_r^n)$ is as follows: the inflow rates into these compartments are $\lambda_r(u_n + i_w^n)$ from transmission, and ωi_w^n from mutation. Hence, we can calculate that 98.7% of macrolide naive resistant infections are originated from transmission of the resistant strain (but one third of those are from overcolonizing a wild-type infected, as we can see from the $i_n^w : u_n$ ratio), and 1.3% are arisen from spontaneous mutations. These fractions were very robust in the sensitivity analysis.

Furthermore, we used the inferred parameters and current state of the system to

compare future scenarios, by solving the system forward in time with the actual parameters, and, for the sake of comparison, assuming that non-eradication purposed macrolide use is being discontinued. We found that (see Supplementary Note 3, and Figure 4 in the main text) discontinuing non-eradication macrolide use would not change significantly the current trend of decreasing prevalence. With the current rate of macrolide use, we predict a further nearly 0.1% increase per year in the rate of clarithromycin resistance in the future, however the discontinuation of non-eradication macrolide use would keep the rate of clarithromycin resistance in the population on a very slowly increasing trajectory. Since there was a difference between males and females in the macrolide consumption rate, have also compared the hypothetical situations where the whole population consumes macrolides at those rates, and we found that higher macrolide consumption rates lead to faster growth in the rate of resistance (Supplementary Fig. 2). We have also investigated the role of κ, and found that the larger κ, the slower the growth in the rate of resistance (Supplementary Fig. 3). The reason is that for larger κ, the bacterial shedding of the resistant type by heteroresistant hosts is smaller, hence there are fewer exposures of susceptibles by the resistant type.

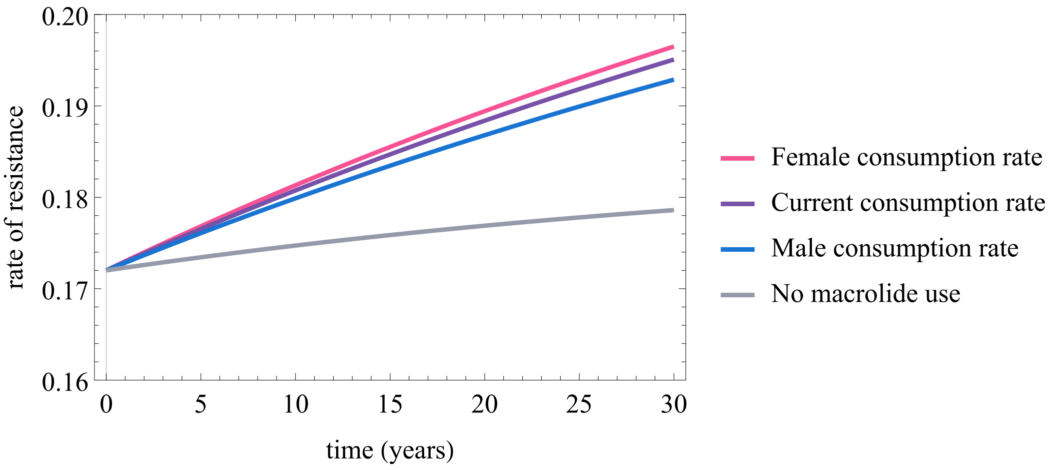

Supplementary Fig. 2: Predicted future rate of resistance in four scenarios: the whole population consume non-eradication purpose macrolide the same rate as females currently do; the whole population consume non-eradication purpose macrolide the same rate as currently do,the whole population consume non-eradication purpose macrolide the same rate as males currently do; the population stop using non-eradication purpose macrolide.

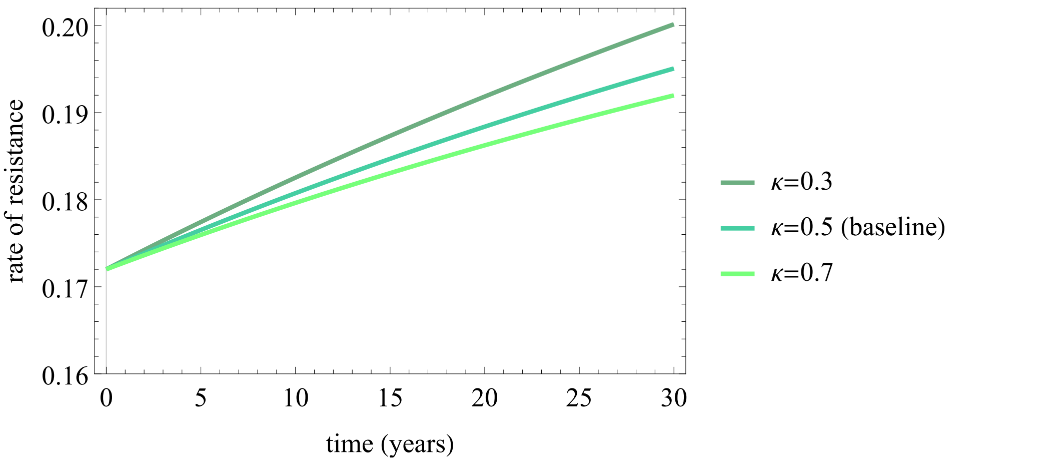

Supplementary Fig. 3: Predicted future rate of resistance for different values of κ.

Supplementary Note 3 : The codes for numerical calculations and simulations

https://www.wolframcloud.com/env/61da20c7-76f5-4fe9-87a6-3174076d6f8c

(*This supplementary note includes the Wolfram Mathematica codes used for the calculations with the mathematical model.*)

```
(*vars=list of model variables*)
vars = {uₙ, iwn, irn, ihn, um, iwm, irm, ihm, uc, iwc, irc, ihc}
```

$\{u_n, i_{wn}, i_{rn}, i_{hn}, u_m, i_{wm}, i_{rm}, i_{hm}, u_c, i_{wc}, i_{rc}, i_{hc}\}$

(*rhs=right hand side of the epidemiological model*)

$$
\begin{aligned}
&\mathrm{rhs} = \{\mu - (\mu + \mathrm{ma})\, u_n - (\lambda_w + \lambda_r)\, u_n, \\
&\lambda_w u_n - i_{wn} * (\mu + \mathrm{ma} + \lambda_r + \theta + \omega) \\
&, \lambda_r u_n - i_{rn} * (\mu + \mathrm{ma} + \lambda_w + \theta) \\
&, \lambda_r i_{wn} + \lambda_w i_{rn} - (\mu + \mathrm{ma} + \theta)\, i_{hn} + \omega * i_{wn} \\
&, -\mu u_m - (\lambda_w + \lambda_r)\, u_m + \mathrm{ma}\, u_n \\
&, \lambda_w u_m - i_{wm} * (\mu + \lambda_r + \theta + \omega) + (1 - \eta)\, \mathrm{ma}\, i_{wn} \\
&, \lambda_r u_m - i_{rm} * (\mu + \lambda_w + \theta) + \mathrm{ma}\, i_{rn} \\
&, \lambda_r i_{wm} + \lambda_w i_{rm} - i_{hm} * (\mu + \theta) + \mathrm{ma}\, i_{hn} + \eta\, \mathrm{ma}\, i_{wn} + \omega\, i_{wm} \\
&, -(\mu + \lambda_r + \lambda_w)\, u_c + \rho_u \theta\, (i_{wn} + i_{wm} + i_{wc}) \\
&\quad, \lambda_w u_c - i_{wc}\, (\mu + \lambda_r + \theta + \omega + \mathrm{ma}\, \eta) + \rho_w \theta\, (i_{wn} + i_{wm} + i_{wc}) \\
&\quad, \lambda_r u_c - i_{rc}\, (\mu + \lambda_w + \theta) + \rho_r \theta\, (i_{wn} + i_{wm} + i_{wc}) + \theta\, (i_{rn} + i_{rm} + i_{rc}) + (\rho_u + \rho_r)\, \theta\, (i_{hn} + i_{hm} + i_{hc}) \\
&\quad, \lambda_r i_{wc} + \lambda_w i_{rc} - i_{hc}\, (\mu + \theta) + (\omega + \mathrm{ma}\, \eta)\, i_{wc} \\
&\quad\ + \rho_h \theta\, (i_{wn} + i_{wm} + i_{wc}) + (\rho_w + \rho_h)\, \theta\, (i_{hn} + i_{hm} + i_{hc}) \\
&\}
\end{aligned}
$$

$$
\begin{aligned}
&\{\mu - (\mathrm{ma} + \mu)\, u_n - u_n\, (\lambda_r + \lambda_w), -i_{wn}\, (\mathrm{ma} + \theta + \mu + \omega + \lambda_r) + u_n \lambda_w, \\
&u_n \lambda_r - i_{rn}\, (\mathrm{ma} + \theta + \mu + \lambda_w), -(\mathrm{ma} + \theta + \mu)\, i_{hn} + \omega\, i_{wn} + i_{wn} \lambda_r + i_{rn} \lambda_w, \\
&-\mu u_m + \mathrm{ma}\, u_n - u_m\, (\lambda_r + \lambda_w), \mathrm{ma}\, (1 - \eta)\, i_{wn} - i_{wm}\, (\theta + \mu + \omega + \lambda_r) + u_m \lambda_w, \\
&\mathrm{ma}\, i_{rn} + u_m \lambda_r - i_{rm}\, (\theta + \mu + \lambda_w), -(\theta + \mu)\, i_{hm} + \mathrm{ma}\, i_{hn} + \omega\, i_{wm} + \mathrm{ma}\, \eta\, i_{wn} + i_{wm} \lambda_r + i_{rm} \lambda_w, \\
&u_c\, (-\mu - \lambda_r - \lambda_w) + \theta\, (i_{wc} + i_{wm} + i_{wn})\, \rho_u, -i_{wc}\, (\mathrm{ma}\, \eta + \theta + \mu + \omega + \lambda_r) + u_c \lambda_w + \theta\, (i_{wc} + i_{wm} + i_{wn})\, \rho_w, \\
&\theta\, (i_{rc} + i_{rm} + i_{rn}) + u_c \lambda_r - i_{rc}\, (\theta + \mu + \lambda_w) + \theta\, (i_{wc} + i_{wm} + i_{wn})\, \rho_r + \theta\, (i_{hc} + i_{hm} + i_{hn})\, (\rho_r + \rho_u), \\
&-(\theta + \mu)\, i_{hc} + (\mathrm{ma}\, \eta + \omega)\, i_{wc} + i_{wc} \lambda_r + i_{rc} \lambda_w + \theta\, (i_{wc} + i_{wm} + i_{wn})\, \rho_h + \theta\, (i_{hc} + i_{hm} + i_{hn})\, (\rho_h + \rho_w)\}
\end{aligned}
$$

```
(*a-priori information*)
μ = 0.013;
ma = 0.00889;
η = 0.129; (*0.12 - 0.14*)
ω = 1.55 * 10 ^ (-5);
i_wn = (2392.0 / 4744) * H;
i_wc = (234.0 / 4744) * H;
i_wm = (1302.0 / 4744) * H;
i_rn = (52.0 / 4744) * H;
i_hn = (88.0 / 4744) * H;
(i_hn + i_hm + i_hc) → (385.0 / 4744) * H;
(i_rn + i_rm + i_rc) → (431.0 / 4744) * H;
ρ_u = 0.92; (*0.88 - 0.96*)
ρ_r = 0.0286; (* *0.5-*1.5 *)
ρ_h = 0.0125; (* *0.5-*1.5 *)
ρ_w = 0.0389; (* *0.5-*1.5 *)
u_m  = 1 - H - u_n - u_c;
i_hc = (247.0 / 4744) * H - i_rc;
i_hm = (429.0 / 4744) * H - i_rm;
i_rm =  (431.0 / 4744) * H - i_rn - i_rc;
H = 0.409;
dH = -0.002; (* *-0.003 - -0.001 *)
θ = ((λ_r + λ_w) (1 - H) - μ * H - dH) / (ρ_u * (i_wn + i_wm + i_wc));
```

```
(*rhs=right hand side of the epidemiological model with such information*)
rhs
```

$$\{0.013 - 0.02189\, u_n - u_n\, (\lambda_r + \lambda_w),$$
$$u_n\, \lambda_w - 0.206224\, (0.0219055 + \lambda_r + 3.20968\, (-0.003317 + 0.591\, (\lambda_r + \lambda_w))),$$
$$u_n\, \lambda_r - 0.00448314\, (0.02189 + \lambda_w + 3.20968\, (-0.003317 + 0.591\, (\lambda_r + \lambda_w))),$$
$$3.19648 \times 10^{-6} + 0.206224\, \lambda_r + 0.00448314\, \lambda_w - 0.00758685\, (0.02189 + 3.20968\, (-0.003317 + 0.591\, (\lambda_r + \lambda_w))),$$
$$-0.013\, (0.591 - u_c - u_n) + 0.00889\, u_n - (0.591 - u_c - u_n)\, (\lambda_r + \lambda_w),$$
$$0.00159683 + (0.591 - u_c - u_n)\, \lambda_w - 0.112251\, (0.0130155 + \lambda_r + 3.20968\, (-0.003317 + 0.591\, (\lambda_r + \lambda_w))),\ 0.0000398551 + (0.591 - u_c - u_n)\, \lambda_r - (0.0326752 - i_{rc})\, (0.013 + \lambda_w + 3.20968\, (-0.003317 + 0.591\, (\lambda_r + \lambda_w))),$$
$$0.000305687 + 0.112251\, \lambda_r + (0.0326752 - i_{rc})\, \lambda_w - (0.00431071 + i_{rc})\, (0.013 + 3.20968\, (-0.003317 + 0.591\, (\lambda_r + \lambda_w))),$$
$$u_c\, (-0.013 - \lambda_r - \lambda_w) + 1.\, (-0.003317 + 0.591\, (\lambda_r + \lambda_w)),$$
$$u_c\, \lambda_w + 0.0422826\, (-0.003317 + 0.591\, (\lambda_r + \lambda_w)) - 0.0201741\, (0.0141623 + \lambda_r + 3.20968\, (-0.003317 + 0.591\, (\lambda_r + \lambda_w))),$$
$$u_c\, \lambda_r + 0.251415\, (-0.003317 + 0.591\, (\lambda_r + \lambda_w)) - i_{rc}\, (0.013 + \lambda_w + 3.20968\, (-0.003317 + 0.591\, (\lambda_r + \lambda_w))),$$
$$0.0000234486 + 0.0201741\, \lambda_r + i_{rc}\, \lambda_w + 0.019063\, (-0.003317 + 0.591\, (\lambda_r + \lambda_w)) - (0.0212949 - i_{rc})\, (0.013 + 3.20968\, (-0.003317 + 0.591\, (\lambda_r + \lambda_w)))\}$$

```
(*rel=relative variations of variables*)
rel = rhs / vars
```

$$\left\{\frac{0.013 - 0.02189\, u_n - u_n (\lambda_r + \lambda_w)}{u_n},\right.$$

$4.84909 (u_n \lambda_w - 0.206224 (0.0219055 + \lambda_r + 3.20968 (-0.003317 + 0.591 (\lambda_r + \lambda_w)))),$

$223.058 (u_n \lambda_r - 0.00448314 (0.02189 + \lambda_w + 3.20968 (-0.003317 + 0.591 (\lambda_r + \lambda_w)))),$

$131.807 \big(3.19648 \times 10^{-6} + 0.206224\, \lambda_r + 0.00448314\, \lambda_w - 0.00758685 (0.02189 + 3.20968 (-0.003317 + 0.591 (\lambda_r + \lambda_w)))\big),$

$(-0.013 (0.591 - u_c - u_n) + 0.00889\, u_n - (0.591 - u_c - u_n) (\lambda_r + \lambda_w)) / (0.591 - u_c - u_n),$

$8.90862 (0.00159683 + (0.591 - u_c - u_n) \lambda_w - 0.112251 (0.0130155 + \lambda_r + 3.20968 (-0.003317 + 0.591 (\lambda_r + \lambda_w)))),$

$(0.0000398551 + (0.591 - u_c - u_n) \lambda_r - (0.0326752 - i_{rc}) (0.013 + \lambda_w + 3.20968 (-0.003317 + 0.591 (\lambda_r + \lambda_w)))) / (0.0326752 - i_{rc}),$

$(0.000305687 + 0.112251\, \lambda_r + (0.0326752 - i_{rc}) \lambda_w - (0.00431071 + i_{rc}) (0.013 + 3.20968 (-0.003317 + 0.591 (\lambda_r + \lambda_w)))) / (0.00431071 + i_{rc}),$

$\frac{1}{u_c} (u_c (-0.013 - \lambda_r - \lambda_w) + 1. (-0.003317 + 0.591 (\lambda_r + \lambda_w))),$

$49.5685 (u_c \lambda_w + 0.0422826 (-0.003317 + 0.591 (\lambda_r + \lambda_w)) - 0.0201741 (0.0141623 + \lambda_r + 3.20968 (-0.003317 + 0.591 (\lambda_r + \lambda_w)))),$

$\frac{1}{i_{rc}} (u_c \lambda_r + 0.251415 (-0.003317 + 0.591 (\lambda_r + \lambda_w)) - i_{rc} (0.013 + \lambda_w + 3.20968 (-0.003317 + 0.591 (\lambda_r + \lambda_w)))), \frac{1}{0.0212949 - i_{rc}}$

$\left.(0.0000234486 + 0.0201741\, \lambda_r + i_{rc} \lambda_w + 0.019063 (-0.003317 + 0.591 (\lambda_r + \lambda_w)) - (0.0212949 - i_{rc}) (0.013 + 3.20968 (-0.003317 + 0.591 (\lambda_r + \lambda_w))))\right\}$

```
(*sums of squares*)
W = Total[rel^2]
```

```
(-0.013 (0.591 - u_c - u_n) + 0.00889 u_n - (0.591 - u_c - u_n) (λ_r + λ_w))^2 / (0.591 - u_c - u_n)^2 +
  (0.013 - 0.02189 u_n - u_n (λ_r + λ_w))^2 / u_n^2 +
  1/u_c^2 (u_c (-0.013 - λ_r - λ_w) + 1. (-0.003317 + 0.591 (λ_r + λ_w)))^2 + 1/(0.0212949 - i_rc)^2
   (0.0000234486 + 0.0201741 λ_r + i_rc λ_w + 0.019063 (-0.003317 + 0.591 (λ_r + λ_w)) -
      (0.0212949 - i_rc) (0.013 + 3.20968 (-0.003317 + 0.591 (λ_r + λ_w))))^2 +
  (0.000305687 + 0.112251 λ_r + (0.0326752 - i_rc) λ_w -
      (0.00431071 + i_rc) (0.013 + 3.20968 (-0.003317 + 0.591 (λ_r + λ_w))))^2 /
   (0.00431071 + i_rc)^2 + 17373.1 (3.19648×10^-6 + 0.206224 λ_r + 0.00448314 λ_w -
      0.00758685 (0.02189 + 3.20968 (-0.003317 + 0.591 (λ_r + λ_w))))^2 +
  79.3635 (0.00159683 + (0.591 - u_c - u_n) λ_w - 0.112251
       (0.0130155 + λ_r + 3.20968 (-0.003317 + 0.591 (λ_r + λ_w))))^2 +
  2457.03 (u_c λ_w + 0.0422826 (-0.003317 + 0.591 (λ_r + λ_w)) -
      0.0201741 (0.0141623 + λ_r + 3.20968 (-0.003317 + 0.591 (λ_r + λ_w))))^2 +
  23.5137 (u_n λ_w - 0.206224 (0.0219055 + λ_r + 3.20968 (-0.003317 + 0.591 (λ_r + λ_w))))^2 +
  (0.0000398551 + (0.591 - u_c - u_n) λ_r -
      (0.0326752 - i_rc) (0.013 + λ_w + 3.20968 (-0.003317 + 0.591 (λ_r + λ_w))))^2 /
   (0.0326752 - i_rc)^2 + 1/i_rc^2 (u_c λ_r + 0.251415 (-0.003317 + 0.591 (λ_r + λ_w)) -
      i_rc (0.013 + λ_w + 3.20968 (-0.003317 + 0.591 (λ_r + λ_w))))^2 +
  49754.9 (u_n λ_r - 0.00448314 (0.02189 + λ_w + 3.20968 (-0.003317 + 0.591 (λ_r + λ_w))))^2
```

```
(*Minimizing W over feasible region with given constraints*)
NMinimize[{W, u_n > 0.0001, u_c > 0.0001, u_n + u_c < 1 - H, λ_r > 0, λ_w > 0,
   0.052 * H - 0.0001 > i_rc > 0.0001, i_rn + i_rc > 0.091 * 0. H - 0.09 * 0. H + 0.0001},
  {λ_r, λ_w, u_n, u_c, i_rc}, Method → {"DifferentialEvolution"}]
```

```
{0.000768398,
 {λ_r → 0.00040911, λ_w → 0.00676164, u_n → 0.390557, u_c → 0.0455673, i_rc → 0.0141463}}
```

```
output = Last[%]
```

```
{λ_r → 0.00040911, λ_w → 0.00676164, u_n → 0.390557, u_c → 0.0455673, i_rc → 0.0141463}
```

```
θ /. output
```

```
0.00295584
```

```
(*{u_n,i_wn,i_rn,i_hn,u_m,i_wm,i_rm,i_hm,u_c,i_wc,i_rc,i_hc}*)
vars /. output
```

```
{0.390557, 0.206224, 0.00448314, 0.00758685, 0.154876, 0.112251,
 0.0185289, 0.018457, 0.0455673, 0.0201741, 0.0141463, 0.00714859}
```

```
Total[vars]
```

```
1.
```

```
(*Share of resistant transmissions of all transmissions*)
λr / (λr + λw) /. output
```

0.0570526

```
(*Origion of primary resistance due to transmission*)
(λr * (un + iwn)) / (λr * (un + iwn) + ω * iwn) /. output
```

0.987077

```
(*calculation of δ*)
δ = (λr (iwn + iwm + iwc + κ (ihn + ihm + ihc))) / (λw ((irn + irn + irn) + (1 - κ) (ihn + ihm + ihc))) /.
   output /. {κ → 0.5}
```

0.715377

```
(*calculation of β*)
β = λw / (iwn + iwm + iwc + κ (ihn + ihm + ihc)) /. output /. κ → 0.5
```

0.0190337

```
(*solving the model with the obtained values*)
{ma, θ, ω, μ, ρh, ρu, ρr, ρw, η, β, δ, κ} =
  {ma, θ, ω, μ, ρh, ρu, ρr, ρw, η, β, δ, κ} /. output /. κ → 0.5;
SystemInitial = vars /. output;
Model = NDSolve[{{uun'[t], iiwn'[t], iirn'[t], iihn'[t], uum'[t], iiwm'[t], iirm'[t],
      iihm'[t], uuc'[t], iiwc'[t], iirc'[t], iihc'[t]} == {μ - (ma + μ) uun[t] -
        (β δ ((1 - κ) (iihc[t] + iihm[t] + iihn[t]) + (iirn[t] + iirc[t] + iirm[t])) +
           β (κ (iihc[t] + iihm[t] + iihn[t]) + iiwc[t] + iiwm[t] + iiwn[t])) uun[t],
      - (ma + θ + μ + ω + β δ ((1 - κ) (iihc[t] + iihm[t] + iihn[t]) + (iirn[t] + iirc[t] + iirm[
                    t]))) iiwn[t] + β (κ (iihc[t] + iihm[t] + iihn[t]) + iiwc[t] + iiwm[t] +
           iiwn[t]) uun[t], -iirn[t] (ma + θ + μ + β (κ (iihc[t] + iihm[t] + iihn[t]) +
              iiwc[t] + iiwm[t] + iiwn[t])) + β δ ((1 - κ) (iihc[t] + iihm[t] + iihn[t]) +
           (iirn[t] + iirc[t] + iirm[t])) uun[t], - (ma + θ + μ) iihn[t] + ω iiwn[t] +
        β δ ((1 - κ) (iihc[t] + iihm[t] + iihn[t]) + (iirn[t] + iirc[t] + iirm[t])) iiwn[t] +
        β iirn[t] (κ (iihc[t] + iihm[t] + iihn[t]) + iiwc[t] + iiwm[t] + iiwn[t]), -μ uum[t] -
        (β δ ((1 - κ) (iihc[t] + iihm[t] + iihn[t]) + (iirn[t] + iirc[t] + iirm[t])) +
           β (κ (iihc[t] + iihm[t] + iihn[t]) + iiwc[t] + iiwm[t] + iiwn[t])) uum[t] +
        ma uun[t], - (θ + μ + ω + β δ ((1 - κ) (iihc[t] + iihm[t] + iihn[t]) +
              (iirn[t] + iirc[t] + iirm[t]))) iiwm[t] + ma (1 - η) iiwn[t] +
        β (κ (iihc[t] + iihm[t] + iihn[t]) + iiwc[t] + iiwm[t] + iiwn[t]) uum[t], ma iirn[t] -
        iirm[t] (θ + μ + β (κ (iihc[t] + iihm[t] + iihn[t]) + iiwc[t] + iiwm[t] + iiwn[t])) +
        β δ ((1 - κ) (iihc[t] + iihm[t] + iihn[t]) + (iirn[t] + iirc[t] + iirm[t])) uum[t],
      - (θ + μ) iihm[t] + ma iihn[t] + ω iiwm[t] +
        β δ ((1 - κ) (iihc[t] + iihm[t] + iihn[t]) + (iirn[t] + iirc[t] + iirm[t])) iiwm[t] + ma
         η iiwn[t] + β iirm[t] (κ (iihc[t] + iihm[t] + iihn[t]) + iiwc[t] + iiwm[t] + iiwn[t]),
      θ ρu (iiwc[t] + iiwm[t] + iiwn[t]) +
```

```
(-μ - β δ ((1 - κ) (ii_hc[t] + ii_hm[t] + ii_hn[t]) + (ii_rn[t] + ii_rc[t] + ii_rm[t])) -
      β (κ (ii_hc[t] + ii_hm[t] + ii_hn[t]) + ii_wc[t] + ii_wm[t] + ii_wn[t])) uu_c[t],
 -(ma η + θ + μ + ω + β δ ((1 - κ) (ii_hc[t] + ii_hm[t] + ii_hn[t]) +
            (ii_rn[t] + ii_rc[t] + ii_rm[t]))) ii_wc[t] + θ ρ_w (ii_wc[t] + ii_wm[t] + ii_wn[t]) +
  β (κ (ii_hc[t] + ii_hm[t] + ii_hn[t]) + ii_wc[t] + ii_wm[t] + ii_wn[t]) uu_c[t],
 θ (ρ_r + ρ_u) (ii_hc[t] + ii_hm[t] + ii_hn[t]) + θ (ii_rc[t] + ii_rm[t] + ii_rn[t]) +
  θ ρ_r (ii_wc[t] + ii_wm[t] + ii_wn[t]) -
  ii_rc[t] (θ + μ + β (κ (ii_hc[t] + ii_hm[t] + ii_hn[t]) + ii_wc[t] + ii_wm[t] + ii_wn[t])) +
  β δ ((1 - κ) (ii_hc[t] + ii_hm[t] + ii_hn[t]) + (ii_rn[t] + ii_rc[t] + ii_rm[t])) uu_c[t],
 -(θ + μ) ii_hc[t] + θ (ρ_h + ρ_w) (ii_hc[t] + ii_hm[t] + ii_hn[t]) + (ma η + ω) ii_wc[t] +
  β δ ((1 - κ) (ii_hc[t] + ii_hm[t] + ii_hn[t]) + (ii_rn[t] + ii_rc[t] + ii_rm[t])) ii_wc[t] +
  θ ρ_h (ii_wc[t] + ii_wm[t] + ii_wn[t]) +
  β ii_rc[t] (κ (ii_hc[t] + ii_hm[t] + ii_hn[t]) + ii_wc[t] + ii_wm[t] + ii_wn[t])},
{uu_n[0], ii_wn[0], ii_rn[0], ii_hn[0], uu_m[0], ii_wm[0], ii_rm[0], ii_hm[0],
  uu_c[0], ii_wc[0], ii_rc[0], ii_hc[0]} == SystemInitial},
{uu_n, ii_wn, ii_rn, ii_hn, uu_m, ii_wm, ii_rm, ii_hm, uu_c, ii_wc, ii_rc, ii_hc}, {t, 0, 1000}];
```

```
(*solving the model with the obtained values but setting macrolide use to 0*)
{ma, θ, ω, μ, ρh, ρu, ρr, ρw, η, β, δ, κ} =
  {ma, θ, ω, μ, ρh, ρu, ρr, ρw, η, β, δ, κ} /. output /. κ → 0.5 /. ma → 0;
SystemInitial = vars /. output;
NoMac = NDSolve[{{uun'[t], iiwn'[t], iirn'[t], iihn'[t], uum'[t], iiwm'[t], iirm'[t],
      iihm'[t], uuc'[t], iiwc'[t], iirc'[t], iihc'[t]} == {μ - (ma + μ) uun[t] -
       (β δ ((1 - κ) (iihc[t] + iihm[t] + iihn[t]) + (iirn[t] + iirc[t] + iirm[t])) +
           β (κ (iihc[t] + iihm[t] + iihn[t]) + iiwc[t] + iiwm[t] + iiwn[t])) uun[t], - (ma + θ +
            μ + ω + β δ ((1 - κ) (iihc[t] + iihm[t] + iihn[t]) + (iirn[t] + iirc[t] + iirm[t])))
         iiwn[t] + β (κ (iihc[t] + iihm[t] + iihn[t]) + iiwc[t] + iiwm[t] + iiwn[t]) uun[t],
      - iirn[t] (ma + θ + μ + β (κ (iihc[t] + iihm[t] + iihn[t]) + iiwc[t] + iiwm[t] + iiwn[t])) +
       β δ ((1 - κ) (iihc[t] + iihm[t] + iihn[t]) + (iirn[t] + iirc[t] + iirm[t])) uun[t],
      - (ma + θ + μ) iihn[t] + ω iiwn[t] +
       β δ ((1 - κ) (iihc[t] + iihm[t] + iihn[t]) + (iirn[t] + iirc[t] + iirm[t])) iiwn[t] +
       β iirn[t] (κ (iihc[t] + iihm[t] + iihn[t]) + iiwc[t] + iiwm[t] + iiwn[t]),
      - μ uum[t] - (β δ ((1 - κ) (iihc[t] + iihm[t] + iihn[t]) + (iirn[t] + iirc[t] + iirm[t])) +
           β (κ (iihc[t] + iihm[t] + iihn[t]) + iiwc[t] + iiwm[t] + iiwn[t])) uum[t] + ma uun[t],
      - (θ + μ + ω + β δ ((1 - κ) (iihc[t] + iihm[t] + iihn[t]) + (iirn[t] + iirc[t] + iirm[t])))
         iiwm[t] + ma (1 - η) iiwn[t] +
       β (κ (iihc[t] + iihm[t] + iihn[t]) + iiwc[t] + iiwm[t] + iiwn[t]) uum[t], ma iirn[t] -
       iirm[t] (θ + μ + β (κ (iihc[t] + iihm[t] + iihn[t]) + iiwc[t] + iiwm[t] + iiwn[t])) +
       β δ ((1 - κ) (iihc[t] + iihm[t] + iihn[t]) + (iirn[t] + iirc[t] + iirm[t])) uum[t],
      - (θ + μ) iihm[t] + ma iihn[t] + ω iiwm[t] +
       β δ ((1 - κ) (iihc[t] + iihm[t] + iihn[t]) + (iirn[t] + iirc[t] + iirm[t])) iiwm[t] +
       ma η iiwn[t] + β iirm[t] (κ (iihc[t] + iihm[t] + iihn[t]) + iiwc[t] + iiwm[t] + iiwn[t]),
      θ ρu (iiwc[t] + iiwm[t] + iiwn[t]) +
       (-μ - β δ ((1 - κ) (iihc[t] + iihm[t] + iihn[t]) + (iirn[t] + iirc[t] + iirm[t])) - β
            (κ (iihc[t] + iihm[t] + iihn[t]) + iiwc[t] + iiwm[t] + iiwn[t])) uuc[t], - (ma η + θ +
            μ + ω + β δ ((1 - κ) (iihc[t] + iihm[t] + iihn[t]) + (iirn[t] + iirc[t] + iirm[t])))
         iiwc[t] + θ ρw (iiwc[t] + iiwm[t] + iiwn[t]) + β (κ (iihc[t] + iihm[t] + iihn[t]) +
           iiwc[t] + iiwm[t] + iiwn[t]) uuc[t], θ (ρr + ρu) (iihc[t] + iihm[t] + iihn[t]) +
       θ (iirc[t] + iirm[t] + iirn[t]) + θ ρr (iiwc[t] + iiwm[t] + iiwn[t]) -
       iirc[t] (θ + μ + β (κ (iihc[t] + iihm[t] + iihn[t]) + iiwc[t] + iiwm[t] + iiwn[t])) +
       β δ ((1 - κ) (iihc[t] + iihm[t] + iihn[t]) + (iirn[t] + iirc[t] + iirm[t])) uuc[t],
      - (θ + μ) iihc[t] + θ (ρh + ρw) (iihc[t] + iihm[t] + iihn[t]) + (ma η + ω) iiwc[t] +
       β δ ((1 - κ) (iihc[t] + iihm[t] + iihn[t]) + (iirn[t] + iirc[t] + iirm[t])) iiwc[t] +
       θ ρh (iiwc[t] + iiwm[t] + iiwn[t]) +
       β iirc[t] (κ (iihc[t] + iihm[t] + iihn[t]) + iiwc[t] + iiwm[t] + iiwn[t])},
    {uun[0], iiwn[0], iirn[0], iihn[0], uum[0], iiwm[0], iirm[0], iihm[0],
      uuc[0], iiwc[0], iirc[0], iihc[0]} == SystemInitial},
   {uun, iiwn, iirn, iihn, uum, iiwm, iirm, iihm, uuc, iiwc, iirc, iihc}, {t, 0, 1000}];

(*solving the model with the lower values from sensitivity analysis*)
{ma, θ, ω, μ, ρh, ρu, ρr, ρw, η, β, δ, κ} =
```

```
{ma, θ, ω, μ, ρ_h, ρ_u, ρ_r, ρ_w, η, β, δ, κ} /. output /. κ → 0.5 /. β → 0.014 /.
   ma → 0.0089 /. δ → 0.58;
SystemInitial = vars /. output;
LowerModel = NDSolve[{{uu_n'[t], ii_wn'[t], ii_rn'[t], ii_hn'[t], uu_m'[t], ii_wm'[t], ii_rm'[t],
      ii_hm'[t], uu_c'[t], ii_wc'[t], ii_rc'[t], ii_hc'[t]} == {μ - (ma + μ) uu_n[t] -
       (β δ ((1 - κ) (ii_hc[t] + ii_hm[t] + ii_hn[t]) + (ii_rn[t] + ii_rc[t] + ii_rm[t])) +
          β (κ (ii_hc[t] + ii_hm[t] + ii_hn[t]) + ii_wc[t] + ii_wm[t] + ii_wn[t])) uu_n[t],
      -(ma + θ + μ + ω + β δ ((1 - κ) (ii_hc[t] + ii_hm[t] + ii_hn[t]) + (ii_rn[t] + ii_rc[t] + ii_rm[
                   t]))) ii_wn[t] + β (κ (ii_hc[t] + ii_hm[t] + ii_hn[t]) + ii_wc[t] + ii_wm[t] +
          ii_wn[t]) uu_n[t], -ii_rn[t] (ma + θ + μ + β (κ (ii_hc[t] + ii_hm[t] + ii_hn[t]) +
              ii_wc[t] + ii_wm[t] + ii_wn[t])) + β δ ((1 - κ) (ii_hc[t] + ii_hm[t] + ii_hn[t]) +
          (ii_rn[t] + ii_rc[t] + ii_rm[t])) uu_n[t], -(ma + θ + μ) ii_hn[t] + ω ii_wn[t] +
       β δ ((1 - κ) (ii_hc[t] + ii_hm[t] + ii_hn[t]) + (ii_rn[t] + ii_rc[t] + ii_rm[t])) ii_wn[t] +
       β ii_rn[t] (κ (ii_hc[t] + ii_hm[t] + ii_hn[t]) + ii_wc[t] + ii_wm[t] + ii_wn[t]), -μ uu_m[t] -
       (β δ ((1 - κ) (ii_hc[t] + ii_hm[t] + ii_hn[t]) + (ii_rn[t] + ii_rc[t] + ii_rm[t])) +
          β (κ (ii_hc[t] + ii_hm[t] + ii_hn[t]) + ii_wc[t] + ii_wm[t] + ii_wn[t])) uu_m[t] +
       ma uu_n[t], -(θ + μ + ω + β δ ((1 - κ) (ii_hc[t] + ii_hm[t] + ii_hn[t]) +
                (ii_rn[t] + ii_rc[t] + ii_rm[t]))) ii_wm[t] + ma (1 - η) ii_wn[t] +
       β (κ (ii_hc[t] + ii_hm[t] + ii_hn[t]) + ii_wc[t] + ii_wm[t] + ii_wn[t]) uu_m[t], ma ii_rn[t] -
       ii_rm[t] (θ + μ + β (κ (ii_hc[t] + ii_hm[t] + ii_hn[t]) + ii_wc[t] + ii_wm[t] + ii_wn[t])) +
       β δ ((1 - κ) (ii_hc[t] + ii_hm[t] + ii_hn[t]) + (ii_rn[t] + ii_rc[t] + ii_rm[t])) uu_m[t],
      -(θ + μ) ii_hm[t] + ma ii_hn[t] + ω ii_wm[t] +
       β δ ((1 - κ) (ii_hc[t] + ii_hm[t] + ii_hn[t]) + (ii_rn[t] + ii_rc[t] + ii_rm[t])) ii_wm[t] + ma
        η ii_wn[t] + β ii_rm[t] (κ (ii_hc[t] + ii_hm[t] + ii_hn[t]) + ii_wc[t] + ii_wm[t] + ii_wn[t]),
      θ ρ_u (ii_wc[t] + ii_wm[t] + ii_wn[t]) +
       (-μ - β δ ((1 - κ) (ii_hc[t] + ii_hm[t] + ii_hn[t]) + (ii_rn[t] + ii_rc[t] + ii_rm[t])) -
          β (κ (ii_hc[t] + ii_hm[t] + ii_hn[t]) + ii_wc[t] + ii_wm[t] + ii_wn[t])) uu_c[t],
      -(ma η + θ + μ + ω + β δ ((1 - κ) (ii_hc[t] + ii_hm[t] + ii_hn[t]) +
                (ii_rn[t] + ii_rc[t] + ii_rm[t]))) ii_wc[t] + θ ρ_w (ii_wc[t] + ii_wm[t] + ii_wn[t]) +
       β (κ (ii_hc[t] + ii_hm[t] + ii_hn[t]) + ii_wc[t] + ii_wm[t] + ii_wn[t]) uu_c[t],
      θ (ρ_r + ρ_u) (ii_hc[t] + ii_hm[t] + ii_hn[t]) + θ (ii_rc[t] + ii_rm[t] + ii_rn[t]) +
       θ ρ_r (ii_wc[t] + ii_wm[t] + ii_wn[t]) -
       ii_rc[t] (θ + μ + β (κ (ii_hc[t] + ii_hm[t] + ii_hn[t]) + ii_wc[t] + ii_wm[t] + ii_wn[t])) +
       β δ ((1 - κ) (ii_hc[t] + ii_hm[t] + ii_hn[t]) + (ii_rn[t] + ii_rc[t] + ii_rm[t])) uu_c[t],
      -(θ + μ) ii_hc[t] + θ (ρ_h + ρ_w) (ii_hc[t] + ii_hm[t] + ii_hn[t]) + (ma η + ω) ii_wc[t] +
       β δ ((1 - κ) (ii_hc[t] + ii_hm[t] + ii_hn[t]) + (ii_rn[t] + ii_rc[t] + ii_rm[t])) ii_wc[t] +
       θ ρ_h (ii_wc[t] + ii_wm[t] + ii_wn[t]) +
       β ii_rc[t] (κ (ii_hc[t] + ii_hm[t] + ii_hn[t]) + ii_wc[t] + ii_wm[t] + ii_wn[t])},
    {uu_n[0], ii_wn[0], ii_rn[0], ii_hn[0], uu_m[0], ii_wm[0], ii_rm[0], ii_hm[0],
      uu_c[0], ii_wc[0], ii_rc[0], ii_hc[0]} == SystemInitial},
   {uu_n, ii_wn, ii_rn, ii_hn, uu_m, ii_wm, ii_rm, ii_hm, uu_c, ii_wc, ii_rc, ii_hc}, {t, 0, 1000}];
```

```
(*solving the model with the higher values from sensitivity analysis*)
{ma, θ, ω, μ, ρ_h, ρ_u, ρ_r, ρ_w, η, β, δ, κ} =
  {ma, θ, ω, μ, ρ_h, ρ_u, ρ_r, ρ_w, η, β, δ, κ} /. output /. κ → 0.5 /. β → 0.024 /.
    ma → 0.0089 /. δ → 0.95;
SystemInitial = vars /. output;
UpperModel = NDSolve[{{uu_n'[t], ii_wn'[t], ii_rn'[t], ii_hn'[t], uu_m'[t], ii_wm'[t], ii_rm'[t],
      ii_hm'[t], uu_c'[t], ii_wc'[t], ii_rc'[t], ii_hc'[t]} == {μ - (ma + μ) uu_n[t] -
        (β δ ((1 - κ) (ii_hc[t] + ii_hm[t] + ii_hn[t]) + (ii_rn[t] + ii_rc[t] + ii_rm[t])) +
            β (κ (ii_hc[t] + ii_hm[t] + ii_hn[t]) + ii_wc[t] + ii_wm[t] + ii_wn[t])) uu_n[t], - (ma + θ +
              μ + ω + β δ ((1 - κ) (ii_hc[t] + ii_hm[t] + ii_hn[t]) + (ii_rn[t] + ii_rc[t] + ii_rm[t])))
          ii_wn[t] + β (κ (ii_hc[t] + ii_hm[t] + ii_hn[t]) + ii_wc[t] + ii_wm[t] + ii_wn[t]) uu_n[t],
      - ii_rn[t] (ma + θ + μ + β (κ (ii_hc[t] + ii_hm[t] + ii_hn[t]) + ii_wc[t] + ii_wm[t] + ii_wn[t])) +
        β δ ((1 - κ) (ii_hc[t] + ii_hm[t] + ii_hn[t]) + (ii_rn[t] + ii_rc[t] + ii_rm[t])) uu_n[t],
      - (ma + θ + μ) ii_hn[t] + ω ii_wn[t] +
        β δ ((1 - κ) (ii_hc[t] + ii_hm[t] + ii_hn[t]) + (ii_rn[t] + ii_rc[t] + ii_rm[t])) ii_wn[t] +
        β ii_rn[t] (κ (ii_hc[t] + ii_hm[t] + ii_hn[t]) + ii_wc[t] + ii_wm[t] + ii_wn[t]),
      - μ uu_m[t] - (β δ ((1 - κ) (ii_hc[t] + ii_hm[t] + ii_hn[t]) + (ii_rn[t] + ii_rc[t] + ii_rm[t])) +
            β (κ (ii_hc[t] + ii_hm[t] + ii_hn[t]) + ii_wc[t] + ii_wm[t] + ii_wn[t])) uu_m[t] + ma uu_n[t],
      - (θ + μ + ω + β δ ((1 - κ) (ii_hc[t] + ii_hm[t] + ii_hn[t]) + (ii_rn[t] + ii_rc[t] + ii_rm[t])))
          ii_wm[t] + ma (1 - η) ii_wn[t] +
        β (κ (ii_hc[t] + ii_hm[t] + ii_hn[t]) + ii_wc[t] + ii_wm[t] + ii_wn[t]) uu_m[t], ma ii_rn[t] -
        ii_rm[t] (θ + μ + β (κ (ii_hc[t] + ii_hm[t] + ii_hn[t]) + ii_wc[t] + ii_wm[t] + ii_wn[t])) +
        β δ ((1 - κ) (ii_hc[t] + ii_hm[t] + ii_hn[t]) + (ii_rn[t] + ii_rc[t] + ii_rm[t])) uu_m[t],
      - (θ + μ) ii_hm[t] + ma ii_hn[t] + ω ii_wm[t] +
        β δ ((1 - κ) (ii_hc[t] + ii_hm[t] + ii_hn[t]) + (ii_rn[t] + ii_rc[t] + ii_rm[t])) ii_wm[t] +
        ma η ii_wn[t] + β ii_rm[t] (κ (ii_hc[t] + ii_hm[t] + ii_hn[t]) + ii_wc[t] + ii_wm[t] + ii_wn[t]),
      θ ρ_u (ii_wc[t] + ii_wm[t] + ii_wn[t]) +
        (- μ - β δ ((1 - κ) (ii_hc[t] + ii_hm[t] + ii_hn[t]) + (ii_rn[t] + ii_rc[t] + ii_rm[t])) - β
             (κ (ii_hc[t] + ii_hm[t] + ii_hn[t]) + ii_wc[t] + ii_wm[t] + ii_wn[t])) uu_c[t], - (ma η + θ +
              μ + ω + β δ ((1 - κ) (ii_hc[t] + ii_hm[t] + ii_hn[t]) + (ii_rn[t] + ii_rc[t] + ii_rm[t])))
          ii_wc[t] + θ ρ_w (ii_wc[t] + ii_wm[t] + ii_wn[t]) + β (κ (ii_hc[t] + ii_hm[t] + ii_hn[t]) +
            ii_wc[t] + ii_wm[t] + ii_wn[t]) uu_c[t], θ (ρ_r + ρ_u) (ii_hc[t] + ii_hm[t] + ii_hn[t]) +
        θ (ii_rc[t] + ii_rm[t] + ii_rn[t]) + θ ρ_r (ii_wc[t] + ii_wm[t] + ii_wn[t]) -
        ii_rc[t] (θ + μ + β (κ (ii_hc[t] + ii_hm[t] + ii_hn[t]) + ii_wc[t] + ii_wm[t] + ii_wn[t])) +
        β δ ((1 - κ) (ii_hc[t] + ii_hm[t] + ii_hn[t]) + (ii_rn[t] + ii_rc[t] + ii_rm[t])) uu_c[t],
      - (θ + μ) ii_hc[t] + θ (ρ_h + ρ_w) (ii_hc[t] + ii_hm[t] + ii_hn[t]) + (ma η + ω) ii_wc[t] +
        β δ ((1 - κ) (ii_hc[t] + ii_hm[t] + ii_hn[t]) + (ii_rn[t] + ii_rc[t] + ii_rm[t])) ii_wc[t] +
        θ ρ_h (ii_wc[t] + ii_wm[t] + ii_wn[t]) +
        β ii_rc[t] (κ (ii_hc[t] + ii_hm[t] + ii_hn[t]) + ii_wc[t] + ii_wm[t] + ii_wn[t])},
    {uu_n[0], ii_wn[0], ii_rn[0], ii_hn[0], uu_m[0], ii_wm[0], ii_rm[0], ii_hm[0],
      uu_c[0], ii_wc[0], ii_rc[0], ii_hc[0]} == SystemInitial},
   {uu_n, ii_wn, ii_rn, ii_hn, uu_m, ii_wm, ii_rm, ii_hm, uu_c, ii_wc, ii_rc, ii_hc}, {t, 0, 1000}];

(*solving the model with the lower values from sensitivity
 analysis without macrolide*) {ma, θ, ω, μ, ρ_h, ρ_u, ρ_r, ρ_w, η, β, δ, κ} =
```

```
{ma, θ, ω, μ, ρ_h, ρ_u, ρ_r, ρ_w, η, β, δ, κ} /. output /. κ → 0.5 /. β → 0.014 /. ma → 0 /.
   δ → 0.58;
SystemInitial = vars /. output;
LowerNoMac = NDSolve[{{uu_n'[t], ii_wn'[t], ii_rn'[t], ii_hn'[t], uu_m'[t], ii_wm'[t], ii_rm'[t],
      ii_hm'[t], uu_c'[t], ii_wc'[t], ii_rc'[t], ii_hc'[t]} == {μ - (ma + μ) uu_n[t] -
       (β δ ((1 - κ) (ii_hc[t] + ii_hm[t] + ii_hn[t]) + (ii_rn[t] + ii_rc[t] + ii_rm[t])) +
          β (κ (ii_hc[t] + ii_hm[t] + ii_hn[t]) + ii_wc[t] + ii_wm[t] + ii_wn[t])) uu_n[t],
      - (ma + θ + μ + ω + β δ ((1 - κ) (ii_hc[t] + ii_hm[t] + ii_hn[t]) + (ii_rn[t] + ii_rc[t] + ii_rm[
                     t]))) ii_wn[t] + β (κ (ii_hc[t] + ii_hm[t] + ii_hn[t]) + ii_wc[t] + ii_wm[t] +
          ii_wn[t]) uu_n[t], -ii_rn[t] (ma + θ + μ + β (κ (ii_hc[t] + ii_hm[t] + ii_hn[t]) +
             ii_wc[t] + ii_wm[t] + ii_wn[t])) + β δ ((1 - κ) (ii_hc[t] + ii_hm[t] + ii_hn[t]) +
          (ii_rn[t] + ii_rc[t] + ii_rm[t])) uu_n[t], - (ma + θ + μ) ii_hn[t] + ω ii_wn[t] +
       β δ ((1 - κ) (ii_hc[t] + ii_hm[t] + ii_hn[t]) + (ii_rn[t] + ii_rc[t] + ii_rm[t])) ii_wn[t] +
       β ii_rn[t] (κ (ii_hc[t] + ii_hm[t] + ii_hn[t]) + ii_wc[t] + ii_wm[t] + ii_wn[t]), -μ uu_m[t] -
       (β δ ((1 - κ) (ii_hc[t] + ii_hm[t] + ii_hn[t]) + (ii_rn[t] + ii_rc[t] + ii_rm[t])) +
          β (κ (ii_hc[t] + ii_hm[t] + ii_hn[t]) + ii_wc[t] + ii_wm[t] + ii_wn[t])) uu_m[t] +
       ma uu_n[t], - (θ + μ + ω + β δ ((1 - κ) (ii_hc[t] + ii_hm[t] + ii_hn[t]) +
                (ii_rn[t] + ii_rc[t] + ii_rm[t]))) ii_wm[t] + ma (1 - η) ii_wn[t] +
       β (κ (ii_hc[t] + ii_hm[t] + ii_hn[t]) + ii_wc[t] + ii_wm[t] + ii_wn[t]) uu_m[t], ma ii_rn[t] -
       ii_rm[t] (θ + μ + β (κ (ii_hc[t] + ii_hm[t] + ii_hn[t]) + ii_wc[t] + ii_wm[t] + ii_wn[t])) +
       β δ ((1 - κ) (ii_hc[t] + ii_hm[t] + ii_hn[t]) + (ii_rn[t] + ii_rc[t] + ii_rm[t])) uu_m[t],
      - (θ + μ) ii_hm[t] + ma ii_hn[t] + ω ii_wm[t] +
       β δ ((1 - κ) (ii_hc[t] + ii_hm[t] + ii_hn[t]) + (ii_rn[t] + ii_rc[t] + ii_rm[t])) ii_wm[t] + ma
        η ii_wn[t] + β ii_rm[t] (κ (ii_hc[t] + ii_hm[t] + ii_hn[t]) + ii_wc[t] + ii_wm[t] + ii_wn[t]),
      θ ρ_u (ii_wc[t] + ii_wm[t] + ii_wn[t]) +
       (-μ - β δ ((1 - κ) (ii_hc[t] + ii_hm[t] + ii_hn[t]) + (ii_rn[t] + ii_rc[t] + ii_rm[t])) -
          β (κ (ii_hc[t] + ii_hm[t] + ii_hn[t]) + ii_wc[t] + ii_wm[t] + ii_wn[t])) uu_c[t],
      - (ma η + θ + μ + ω + β δ ((1 - κ) (ii_hc[t] + ii_hm[t] + ii_hn[t]) +
                (ii_rn[t] + ii_rc[t] + ii_rm[t]))) ii_wc[t] + θ ρ_w (ii_wc[t] + ii_wm[t] + ii_wn[t]) +
       β (κ (ii_hc[t] + ii_hm[t] + ii_hn[t]) + ii_wc[t] + ii_wm[t] + ii_wn[t]) uu_c[t],
      θ (ρ_r + ρ_u) (ii_hc[t] + ii_hm[t] + ii_hn[t]) + θ (ii_rc[t] + ii_rm[t] + ii_rn[t]) +
       θ ρ_r (ii_wc[t] + ii_wm[t] + ii_wn[t]) -
       ii_rc[t] (θ + μ + β (κ (ii_hc[t] + ii_hm[t] + ii_hn[t]) + ii_wc[t] + ii_wm[t] + ii_wn[t])) +
       β δ ((1 - κ) (ii_hc[t] + ii_hm[t] + ii_hn[t]) + (ii_rn[t] + ii_rc[t] + ii_rm[t])) uu_c[t],
      - (θ + μ) ii_hc[t] + θ (ρ_h + ρ_w) (ii_hc[t] + ii_hm[t] + ii_hn[t]) + (ma η + ω) ii_wc[t] +
       β δ ((1 - κ) (ii_hc[t] + ii_hm[t] + ii_hn[t]) + (ii_rn[t] + ii_rc[t] + ii_rm[t])) ii_wc[t] +
       θ ρ_h (ii_wc[t] + ii_wm[t] + ii_wn[t]) +
       β ii_rc[t] (κ (ii_hc[t] + ii_hm[t] + ii_hn[t]) + ii_wc[t] + ii_wm[t] + ii_wn[t])},
    {uu_n[0], ii_wn[0], ii_rn[0], ii_hn[0], uu_m[0], ii_wm[0], ii_rm[0], ii_hm[0],
      uu_c[0], ii_wc[0], ii_rc[0], ii_hc[0]} == SystemInitial},
   {uu_n, ii_wn, ii_rn, ii_hn, uu_m, ii_wm, ii_rm, ii_hm, uu_c, ii_wc, ii_rc, ii_hc}, {t, 0, 1000}];
```

```
(*solving the model with the higher values from sensitivity
 analysis without macrolid*) {ma, θ, ω, μ, ρ_h, ρ_u, ρ_r, ρ_w, η, β, δ, κ} =
  {ma, θ, ω, μ, ρ_h, ρ_u, ρ_r, ρ_w, η, β, δ, κ} /. output /. κ → 0.5 /. β → 0.024 /. ma → 0 /.
   δ → 0.95;
SystemInitial = vars /. output;
UpperNoMac = NDSolve[{{uu_n'[t], ii_wn'[t], ii_rn'[t], ii_hn'[t], uu_m'[t], ii_wm'[t], ii_rm'[t],
      ii_hm'[t], uu_c'[t], ii_wc'[t], ii_rc'[t], ii_hc'[t]} == {μ - (ma + μ) uu_n[t] -
        (β δ ((1 - κ) (ii_hc[t] + ii_hm[t] + ii_hn[t]) + (ii_rn[t] + ii_rc[t] + ii_rm[t])) +
            β (κ (ii_hc[t] + ii_hm[t] + ii_hn[t]) + ii_wc[t] + ii_wm[t] + ii_wn[t])) uu_n[t], - (ma + θ +
             μ + ω + β δ ((1 - κ) (ii_hc[t] + ii_hm[t] + ii_hn[t]) + (ii_rn[t] + ii_rc[t] + ii_rm[t])))
          ii_wn[t] + β (κ (ii_hc[t] + ii_hm[t] + ii_hn[t]) + ii_wc[t] + ii_wm[t] + ii_wn[t]) uu_n[t],
       - ii_rn[t] (ma + θ + μ + β (κ (ii_hc[t] + ii_hm[t] + ii_hn[t]) + ii_wc[t] + ii_wm[t] + ii_wn[t])) +
        β δ ((1 - κ) (ii_hc[t] + ii_hm[t] + ii_hn[t]) + (ii_rn[t] + ii_rc[t] + ii_rm[t])) uu_n[t],
       - (ma + θ + μ) ii_hn[t] + ω ii_wn[t] +
        β δ ((1 - κ) (ii_hc[t] + ii_hm[t] + ii_hn[t]) + (ii_rn[t] + ii_rc[t] + ii_rm[t])) ii_wn[t] +
        β ii_rn[t] (κ (ii_hc[t] + ii_hm[t] + ii_hn[t]) + ii_wc[t] + ii_wm[t] + ii_wn[t]),
       - μ uu_m[t] - (β δ ((1 - κ) (ii_hc[t] + ii_hm[t] + ii_hn[t]) + (ii_rn[t] + ii_rc[t] + ii_rm[t])) +
            β (κ (ii_hc[t] + ii_hm[t] + ii_hn[t]) + ii_wc[t] + ii_wm[t] + ii_wn[t])) uu_m[t] + ma uu_n[t],
       - (θ + μ + ω + β δ ((1 - κ) (ii_hc[t] + ii_hm[t] + ii_hn[t]) + (ii_rn[t] + ii_rc[t] + ii_rm[t])))
          ii_wm[t] + ma (1 - η) ii_wn[t] +
        β (κ (ii_hc[t] + ii_hm[t] + ii_hn[t]) + ii_wc[t] + ii_wm[t] + ii_wn[t]) uu_m[t], ma ii_rn[t] -
        ii_rm[t] (θ + μ + β (κ (ii_hc[t] + ii_hm[t] + ii_hn[t]) + ii_wc[t] + ii_wm[t] + ii_wn[t])) +
        β δ ((1 - κ) (ii_hc[t] + ii_hm[t] + ii_hn[t]) + (ii_rn[t] + ii_rc[t] + ii_rm[t])) uu_m[t],
       - (θ + μ) ii_hm[t] + ma ii_hn[t] + ω ii_wm[t] +
        β δ ((1 - κ) (ii_hc[t] + ii_hm[t] + ii_hn[t]) + (ii_rn[t] + ii_rc[t] + ii_rm[t])) ii_wm[t] +
        ma η ii_wn[t] + β ii_rm[t] (κ (ii_hc[t] + ii_hm[t] + ii_hn[t]) + ii_wc[t] + ii_wm[t] + ii_wn[t]),
       θ ρ_u (ii_wc[t] + ii_wm[t] + ii_wn[t]) +
        (- μ - β δ ((1 - κ) (ii_hc[t] + ii_hm[t] + ii_hn[t]) + (ii_rn[t] + ii_rc[t] + ii_rm[t])) - β
             (κ (ii_hc[t] + ii_hm[t] + ii_hn[t]) + ii_wc[t] + ii_wm[t] + ii_wn[t])) uu_c[t], - (ma η + θ +
             μ + ω + β δ ((1 - κ) (ii_hc[t] + ii_hm[t] + ii_hn[t]) + (ii_rn[t] + ii_rc[t] + ii_rm[t])))
          ii_wc[t] + θ ρ_w (ii_wc[t] + ii_wm[t] + ii_wn[t]) + β (κ (ii_hc[t] + ii_hm[t] + ii_hn[t]) +
             ii_wc[t] + ii_wm[t] + ii_wn[t]) uu_c[t], θ (ρ_r + ρ_u) (ii_hc[t] + ii_hm[t] + ii_hn[t]) +
        θ (ii_rc[t] + ii_rm[t] + ii_rn[t]) + θ ρ_r (ii_wc[t] + ii_wm[t] + ii_wn[t]) -
        ii_rc[t] (θ + μ + β (κ (ii_hc[t] + ii_hm[t] + ii_hn[t]) + ii_wc[t] + ii_wm[t] + ii_wn[t])) +
        β δ ((1 - κ) (ii_hc[t] + ii_hm[t] + ii_hn[t]) + (ii_rn[t] + ii_rc[t] + ii_rm[t])) uu_c[t],
       - (θ + μ) ii_hc[t] + θ (ρ_h + ρ_w) (ii_hc[t] + ii_hm[t] + ii_hn[t]) + (ma η + ω) ii_wc[t] +
        β δ ((1 - κ) (ii_hc[t] + ii_hm[t] + ii_hn[t]) + (ii_rn[t] + ii_rc[t] + ii_rm[t])) ii_wc[t] +
        θ ρ_h (ii_wc[t] + ii_wm[t] + ii_wn[t]) +
        β ii_rc[t] (κ (ii_hc[t] + ii_hm[t] + ii_hn[t]) + ii_wc[t] + ii_wm[t] + ii_wn[t])},
    {uu_n[0], ii_wn[0], ii_rn[0], ii_hn[0], uu_m[0], ii_wm[0], ii_rm[0], ii_hm[0],
      uu_c[0], ii_wc[0], ii_rc[0], ii_hc[0]} == SystemInitial},
   {uu_n, ii_wn, ii_rn, ii_hn, uu_m, ii_wm, ii_rm, ii_hm, uu_c, ii_wc, ii_rc, ii_hc}, {t, 0, 1000}];
```

```
plotuncertainty =
 Plot[{Evaluate[((iirn[t] + iirm[t] + iirc[t] + iihn[t] + iihm[t] + iihc[t]) /
       (1 - uun[t] - uum[t] - uuc[t])) /. Model],
   Evaluate[(iirn[t] + iirm[t] + iirc[t] + iihn[t] + iihm[t] + iihc[t]) /
      (1 - uun[t] - uum[t] - uuc[t]) /. NoMac],
   Evaluate[(iirn[t] + iirm[t] + iirc[t] + iihn[t] + iihm[t] + iihc[t]) /
      (1 - uun[t] - uum[t] - uuc[t]) /. LowerModel],
   Evaluate[(iirn[t] + iirm[t] + iirc[t] + iihn[t] + iihm[t] + iihc[t]) /
      (1 - uun[t] - uum[t] - uuc[t]) /. UpperModel],
   Evaluate[(iirn[t] + iirm[t] + iirc[t] + iihn[t] + iihm[t] + iihc[t]) /
      (1 - uun[t] - uum[t] - uuc[t]) /. LowerNoMac],
   Evaluate[(iirn[t] + iirm[t] + iirc[t] + iihn[t] + iihm[t] + iihc[t]) /
      (1 - uun[t] - uum[t] - uuc[t]) /. UpperNoMac]},
  {t, 0, 30}, PlotRange → {0.165, 0.22}, PlotStyle →
   {{ColorData["Crayola"]["Scarlet"], Thickness[0.0075]},
    {ColorData["Crayola"]["PineGreen"], Thickness[0.0075]},
    {ColorData["Crayola"]["Scarlet"], Thickness[0.001]},
    {ColorData["Crayola"]["Scarlet"], Thickness[0.001]},
    {ColorData["Crayola"]["PineGreen"], Thickness[0.001]},
    {ColorData["Crayola"]["PineGreen"], Thickness[0.001]}},
  PlotLegends → {"Rate of resistance (current consumption)",
    "Rate of resistance (without macrolide)"},
  FrameLabel → {"time (years)", "rate of resistance"},
  LabelStyle → Directive[FontFamily → "Times", Black, FontSize → 14],
  PlotTheme → "Scientific", Filling → {3 → {4}, 5 → {6}},
  FillingStyle → Opacity[0.1]]
```

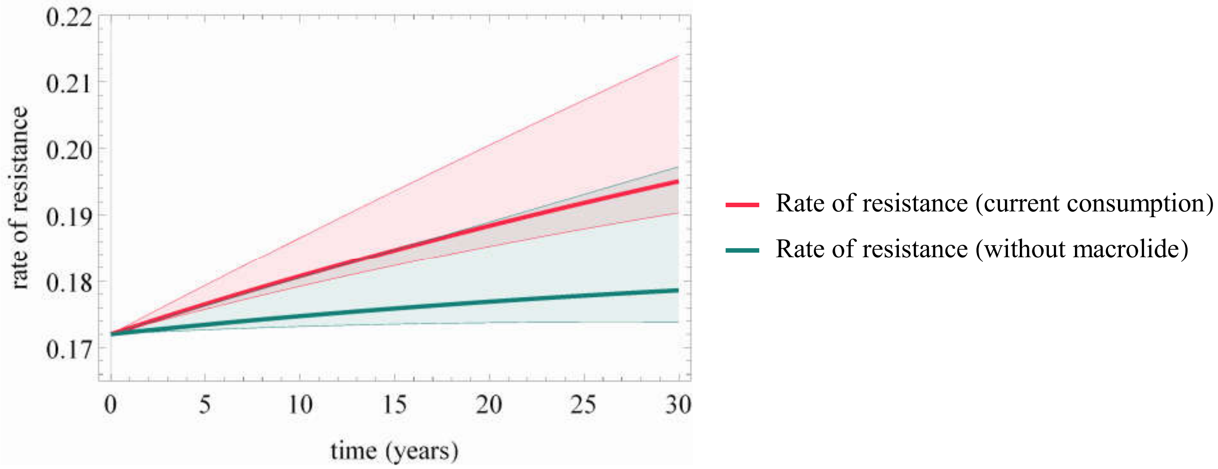


```
(*SetDirectory[NotebookDirectory[]];
Export["uncertainty.pdf",plotuncertainty,"pdf"]*)
```

```
{ma, θ, ω, μ, ρh, ρu, ρr, ρw, η, β, δ, κ} =
  {ma, θ, ω, μ, ρh, ρu, ρr, ρw, η, β, δ, κ} /. output /. β -> 0.019033717154126016` /.
```

```
    δ -> 0.7153772780684298` /. κ → 0.5 /. ma → 0.00757;
SystemInitial = vars /. output;
MaleMac = NDSolve[{{uu_n'[t], ii_wn'[t], ii_rn'[t], ii_hn'[t], uu_m'[t], ii_wm'[t], ii_rm'[t],
     ii_hm'[t], uu_c'[t], ii_wc'[t], ii_rc'[t], ii_hc'[t]} == {μ - (ma + μ) uu_n[t] -
      (β δ ((1 - κ) (ii_hc[t] + ii_hm[t] + ii_hn[t]) + (ii_rn[t] + ii_rc[t] + ii_rm[t])) +
         β (κ (ii_hc[t] + ii_hm[t] + ii_hn[t]) + ii_wc[t] + ii_wm[t] + ii_wn[t])) uu_n[t],
     - (ma + θ + μ + ω + β δ ((1 - κ) (ii_hc[t] + ii_hm[t] + ii_hn[t]) + (ii_rn[t] + ii_rc[t] + ii_rm[
                t]))) ii_wn[t] + β (κ (ii_hc[t] + ii_hm[t] + ii_hn[t]) + ii_wc[t] + ii_wm[t] +
         ii_wn[t]) uu_n[t], - ii_rn[t] (ma + θ + μ + β (κ (ii_hc[t] + ii_hm[t] + ii_hn[t]) +
            ii_wc[t] + ii_wm[t] + ii_wn[t])) + β δ ((1 - κ) (ii_hc[t] + ii_hm[t] + ii_hn[t]) +
         (ii_rn[t] + ii_rc[t] + ii_rm[t])) uu_n[t], - (ma + θ + μ) ii_hn[t] + ω ii_wn[t] +
      β δ ((1 - κ) (ii_hc[t] + ii_hm[t] + ii_hn[t]) + (ii_rn[t] + ii_rc[t] + ii_rm[t])) ii_wn[t] +
      β ii_rn[t] (κ (ii_hc[t] + ii_hm[t] + ii_hn[t]) + ii_wc[t] + ii_wm[t] + ii_wn[t]), - μ uu_m[t] -
      (β δ ((1 - κ) (ii_hc[t] + ii_hm[t] + ii_hn[t]) + (ii_rn[t] + ii_rc[t] + ii_rm[t])) +
         β (κ (ii_hc[t] + ii_hm[t] + ii_hn[t]) + ii_wc[t] + ii_wm[t] + ii_wn[t])) uu_m[t] +
      ma uu_n[t], - (θ + μ + ω + β δ ((1 - κ) (ii_hc[t] + ii_hm[t] + ii_hn[t]) +
              (ii_rn[t] + ii_rc[t] + ii_rm[t]))) ii_wm[t] + ma (1 - η) ii_wn[t] +
      β (κ (ii_hc[t] + ii_hm[t] + ii_hn[t]) + ii_wc[t] + ii_wm[t] + ii_wn[t]) uu_m[t], ma ii_rn[t] -
      ii_rm[t] (θ + μ + β (κ (ii_hc[t] + ii_hm[t] + ii_hn[t]) + ii_wc[t] + ii_wm[t] + ii_wn[t])) +
      β δ ((1 - κ) (ii_hc[t] + ii_hm[t] + ii_hn[t]) + (ii_rn[t] + ii_rc[t] + ii_rm[t])) uu_m[t],
     - (θ + μ) ii_hm[t] + ma ii_hn[t] + ω ii_wm[t] +
      β δ ((1 - κ) (ii_hc[t] + ii_hm[t] + ii_hn[t]) + (ii_rn[t] + ii_rc[t] + ii_rm[t])) ii_wm[t] + ma
       η ii_wn[t] + β ii_rm[t] (κ (ii_hc[t] + ii_hm[t] + ii_hn[t]) + ii_wc[t] + ii_wm[t] + ii_wn[t]),
     θ ρ_u (ii_wc[t] + ii_wm[t] + ii_wn[t]) +
      (- μ - β δ ((1 - κ) (ii_hc[t] + ii_hm[t] + ii_hn[t]) + (ii_rn[t] + ii_rc[t] + ii_rm[t])) -
         β (κ (ii_hc[t] + ii_hm[t] + ii_hn[t]) + ii_wc[t] + ii_wm[t] + ii_wn[t])) uu_c[t],
     - (ma η + θ + μ + ω + β δ ((1 - κ) (ii_hc[t] + ii_hm[t] + ii_hn[t]) +
              (ii_rn[t] + ii_rc[t] + ii_rm[t]))) ii_wc[t] + θ ρ_w (ii_wc[t] + ii_wm[t] + ii_wn[t]) +
      β (κ (ii_hc[t] + ii_hm[t] + ii_hn[t]) + ii_wc[t] + ii_wm[t] + ii_wn[t]) uu_c[t],
     θ (ρ_r + ρ_u) (ii_hc[t] + ii_hm[t] + ii_hn[t]) + θ (ii_rc[t] + ii_rm[t] + ii_rn[t]) +
      θ ρ_r (ii_wc[t] + ii_wm[t] + ii_wn[t]) -
      ii_rc[t] (θ + μ + β (κ (ii_hc[t] + ii_hm[t] + ii_hn[t]) + ii_wc[t] + ii_wm[t] + ii_wn[t])) +
      β δ ((1 - κ) (ii_hc[t] + ii_hm[t] + ii_hn[t]) + (ii_rn[t] + ii_rc[t] + ii_rm[t])) uu_c[t],
     - (θ + μ) ii_hc[t] + θ (ρ_h + ρ_w) (ii_hc[t] + ii_hm[t] + ii_hn[t]) + (ma η + ω) ii_wc[t] +
      β δ ((1 - κ) (ii_hc[t] + ii_hm[t] + ii_hn[t]) + (ii_rn[t] + ii_rc[t] + ii_rm[t])) ii_wc[t] +
      θ ρ_h (ii_wc[t] + ii_wm[t] + ii_wn[t]) +
      β ii_rc[t] (κ (ii_hc[t] + ii_hm[t] + ii_hn[t]) + ii_wc[t] + ii_wm[t] + ii_wn[t])},
   {uu_n[0], ii_wn[0], ii_rn[0], ii_hn[0], uu_m[0], ii_wm[0], ii_rm[0], ii_hm[0],
     uu_c[0], ii_wc[0], ii_rc[0], ii_hc[0]} == SystemInitial},
  {uu_n, ii_wn, ii_rn, ii_hn, uu_m, ii_wm, ii_rm, ii_hm, uu_c, ii_wc, ii_rc, ii_hc}, {t, 0, 1000}];
```

```
{ma, θ, ω, μ, ρ_h, ρ_u, ρ_r, ρ_w, η, β, δ, κ} =
  {ma, θ, ω, μ, ρ_h, ρ_u, ρ_r, ρ_w, η, β, δ, κ} /. output /. β -> 0.019033717154126016` /.
```

```
    δ -> 0.7153772780684298` /. κ → 0.5 /. ma → 0.00977;
SystemInitial = vars /. output;
FemaleMac = NDSolve[{{uu_n′[t], ii_wn′[t], ii_rn′[t], ii_hn′[t], uu_m′[t], ii_wm′[t], ii_rm′[t],
    ii_hm′[t], uu_c′[t], ii_wc′[t], ii_rc′[t], ii_hc′[t]} == {μ - (ma + μ) uu_n[t] -
     (β δ ((1 - κ) (ii_hc[t] + ii_hm[t] + ii_hn[t]) + (ii_rn[t] + ii_rc[t] + ii_rm[t])) +
        β (κ (ii_hc[t] + ii_hm[t] + ii_hn[t]) + ii_wc[t] + ii_wm[t] + ii_wn[t])) uu_n[t],
    -(ma + θ + μ + ω + β δ ((1 - κ) (ii_hc[t] + ii_hm[t] + ii_hn[t]) + (ii_rn[t] + ii_rc[t] + ii_rm[
              t]))) ii_wn[t] + β (κ (ii_hc[t] + ii_hm[t] + ii_hn[t]) + ii_wc[t] + ii_wm[t] +
        ii_wn[t]) uu_n[t], -ii_rn[t] (ma + θ + μ + β (κ (ii_hc[t] + ii_hm[t] + ii_hn[t]) +
           ii_wc[t] + ii_wm[t] + ii_wn[t])) + β δ ((1 - κ) (ii_hc[t] + ii_hm[t] + ii_hn[t]) +
        (ii_rn[t] + ii_rc[t] + ii_rm[t])) uu_n[t], -(ma + θ + μ) ii_hn[t] + ω ii_wn[t] +
     β δ ((1 - κ) (ii_hc[t] + ii_hm[t] + ii_hn[t]) + (ii_rn[t] + ii_rc[t] + ii_rm[t])) ii_wn[t] +
     β ii_rn[t] (κ (ii_hc[t] + ii_hm[t] + ii_hn[t]) + ii_wc[t] + ii_wm[t] + ii_wn[t]), -μ uu_m[t] -
     (β δ ((1 - κ) (ii_hc[t] + ii_hm[t] + ii_hn[t]) + (ii_rn[t] + ii_rc[t] + ii_rm[t])) +
        β (κ (ii_hc[t] + ii_hm[t] + ii_hn[t]) + ii_wc[t] + ii_wm[t] + ii_wn[t])) uu_m[t] +
     ma uu_n[t], -(θ + μ + ω + β δ ((1 - κ) (ii_hc[t] + ii_hm[t] + ii_hn[t]) +
           (ii_rn[t] + ii_rc[t] + ii_rm[t]))) ii_wm[t] + ma (1 - η) ii_wn[t] +
     β (κ (ii_hc[t] + ii_hm[t] + ii_hn[t]) + ii_wc[t] + ii_wm[t] + ii_wn[t]) uu_m[t], ma ii_rn[t] -
     ii_rm[t] (θ + μ + β (κ (ii_hc[t] + ii_hm[t] + ii_hn[t]) + ii_wc[t] + ii_wm[t] + ii_wn[t])) +
     β δ ((1 - κ) (ii_hc[t] + ii_hm[t] + ii_hn[t]) + (ii_rn[t] + ii_rc[t] + ii_rm[t])) uu_m[t],
    -(θ + μ) ii_hm[t] + ma ii_hn[t] + ω ii_wm[t] +
     β δ ((1 - κ) (ii_hc[t] + ii_hm[t] + ii_hn[t]) + (ii_rn[t] + ii_rc[t] + ii_rm[t])) ii_wm[t] + ma
      η ii_wn[t] + β ii_rm[t] (κ (ii_hc[t] + ii_hm[t] + ii_hn[t]) + ii_wc[t] + ii_wm[t] + ii_wn[t]),
    θ ρ_u (ii_wc[t] + ii_wm[t] + ii_wn[t]) +
     (-μ - β δ ((1 - κ) (ii_hc[t] + ii_hm[t] + ii_hn[t]) + (ii_rn[t] + ii_rc[t] + ii_rm[t])) -
        β (κ (ii_hc[t] + ii_hm[t] + ii_hn[t]) + ii_wc[t] + ii_wm[t] + ii_wn[t])) uu_c[t],
    -(ma η + θ + μ + ω + β δ ((1 - κ) (ii_hc[t] + ii_hm[t] + ii_hn[t]) +
           (ii_rn[t] + ii_rc[t] + ii_rm[t]))) ii_wc[t] + θ ρ_w (ii_wc[t] + ii_wm[t] + ii_wn[t]) +
     β (κ (ii_hc[t] + ii_hm[t] + ii_hn[t]) + ii_wc[t] + ii_wm[t] + ii_wn[t]) uu_c[t],
    θ (ρ_r + ρ_u) (ii_hc[t] + ii_hm[t] + ii_hn[t]) + θ (ii_rc[t] + ii_rm[t] + ii_rn[t]) +
     θ ρ_r (ii_wc[t] + ii_wm[t] + ii_wn[t]) -
     ii_rc[t] (θ + μ + β (κ (ii_hc[t] + ii_hm[t] + ii_hn[t]) + ii_wc[t] + ii_wm[t] + ii_wn[t])) +
     β δ ((1 - κ) (ii_hc[t] + ii_hm[t] + ii_hn[t]) + (ii_rn[t] + ii_rc[t] + ii_rm[t])) uu_c[t],
    -(θ + μ) ii_hc[t] + θ (ρ_h + ρ_w) (ii_hc[t] + ii_hm[t] + ii_hn[t]) + (ma η + ω) ii_wc[t] +
     β δ ((1 - κ) (ii_hc[t] + ii_hm[t] + ii_hn[t]) + (ii_rn[t] + ii_rc[t] + ii_rm[t])) ii_wc[t] +
     θ ρ_h (ii_wc[t] + ii_wm[t] + ii_wn[t]) +
     β ii_rc[t] (κ (ii_hc[t] + ii_hm[t] + ii_hn[t]) + ii_wc[t] + ii_wm[t] + ii_wn[t])},
  {uu_n[0], ii_wn[0], ii_rn[0], ii_hn[0], uu_m[0], ii_wm[0], ii_rm[0], ii_hm[0],
    uu_c[0], ii_wc[0], ii_rc[0], ii_hc[0]} == SystemInitial},
 {uu_n, ii_wn, ii_rn, ii_hn, uu_m, ii_wm, ii_rm, ii_hm, uu_c, ii_wc, ii_rc, ii_hc}, {t, 0, 1000}];
```

```
plotmacrate = Plot[{Evaluate[(iirn[t] + iirm[t] + iirc[t] + iihn[t] + iihm[t] + iihc[t]) /
      (1 - uun[t] - uum[t] - uuc[t]) /. FemaleMac],
    Evaluate[((iirn[t] + iirm[t] + iirc[t] + iihn[t] + iihm[t] + iihc[t]) /
       (1 - uun[t] - uum[t] - uuc[t])) /. Model],
    Evaluate[(iirn[t] + iirm[t] + iirc[t] + iihn[t] + iihm[t] + iihc[t]) /
      (1 - uun[t] - uum[t] - uuc[t]) /. MaleMac],
    Evaluate[(iirn[t] + iirm[t] + iirc[t] + iihn[t] + iihm[t] + iihc[t]) /
      (1 - uun[t] - uum[t] - uuc[t]) /. NoMac]}, {t, 0, 30}, PlotRange → {0.16, 0.20},
   PlotStyle → {{ColorData["Crayola"]["VioletRed"], Thickness[0.0075]},
     {ColorData["Crayola"]["RoyalPurple"], Thickness[0.0075]},
     {ColorData["Crayola"]["NavyBlue"], Thickness[0.0075]},
     {ColorData["Crayola"]["Manatee"], Thickness[0.0075]}},
   PlotLegends → {"Female consumption rate", "Current consumption rate",
     "Male consumption rate", "No macrolide use"},
   FrameLabel → {"time (years)", "rate of resistance"},
   LabelStyle → Directive[FontFamily → "Times", Black, FontSize → 14],
   PlotTheme -> "Scientific"]
```

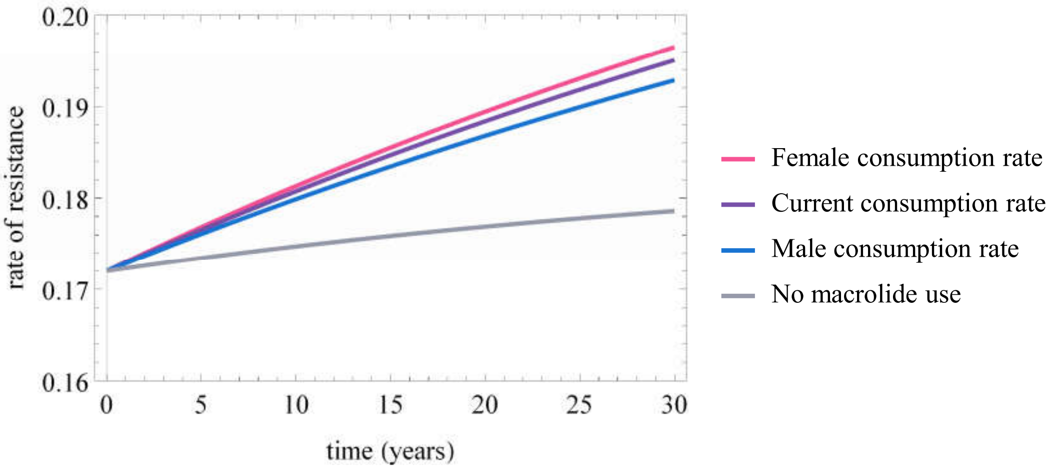

```
Evaluate[((iirn[t] + iirm[t] + iirc[t] + iihn[t] + iihm[t] + iihc[t]) /
      (1 - uun[t] - uum[t] - uuc[t]))] /. Model /. t → 0
Evaluate[((iirn[t] + iirm[t] + iirc[t] + iihn[t] + iihm[t] + iihc[t]) /
      (1 - uun[t] - uum[t] - uuc[t]))] /. Model /. t → 1
Evaluate[((iirn[t] + iirm[t] + iirc[t] + iihn[t] + iihm[t] + iihc[t]) /
      (1 - uun[t] - uum[t] - uuc[t]))] /. Model /. t → 10
Evaluate[((iirn[t] + iirm[t] + iirc[t] + iihn[t] + iihm[t] + iihc[t]) /
      (1 - uun[t] - uum[t] - uuc[t]))] /. Model /. t → 20
Evaluate[((iirn[t] + iirm[t] + iirc[t] + iihn[t] + iihm[t] + iihc[t]) /
      (1 - uun[t] - uum[t] - uuc[t]))] /. Model /. t → 30
```

{0.172007}

{0.172937}

{0.180752}

{0.188385}

{0.195074}

```
(*SetDirectory[NotebookDirectory[]];
Export["macrate.pdf",plotmacrate,"pdf"]*)
```

```
output
vars /. output
(*calculation of δlowkappa*) κ = 0.3;
δlκ = (λr (iwn + iwm + iwc + κ (ihn + ihm + ihc))) /
    (λw ((irn + irn + irn) + (1 - κ) (ihn + ihm + ihc))) /. output
(*calculation of δhighkappa*) κ = 0.7;
δhκ = (λr (iwn + iwm + iwc + κ (ihn + ihm + ihc))) /
    (λw ((irn + irn + irn) + (1 - κ) (ihn + ihm + ihc))) /. output
(*calculation of βlowkappa*) κ = 0.3;
βlκ = λw / (iwn + iwm + iwc + κ (ihn + ihm + ihc)) /. output
(*calculation of βhighkappa*) κ = 0.7;
βhκ = λw / (iwn + iwm + iwc + κ (ihn + ihm + ihc)) /. output
```

{λ_r → 0.00040911, λ_w → 0.00676164, u_n → 0.390557, u_c → 0.0455673, i_{rc} → 0.0141463}

{0.390557, 0.206224, 0.00448314, 0.00758685, 0.154876, 0.112251,
 0.0185289, 0.018457, 0.0455673, 0.0201741, 0.0141463, 0.00714859}

0.574971

0.935425

0.0193962

0.0186846

```
κ = 0.3; {ma, θ, ω, μ, ρh, ρu, ρr, ρw, η, β, δ, κ} =
  {ma, θ, ω, μ, ρh, ρu, ρr, ρw, η, βlκ, δlκ, κ} /. output /. ma -> 0.00889;
```

```
SystemInitial = vars /. output;
LowKappa = NDSolve[{{uu_n'[t], ii_wn'[t], ii_rn'[t], ii_hn'[t], uu_m'[t], ii_wm'[t], ii_rm'[t],
      ii_hm'[t], uu_c'[t], ii_wc'[t], ii_rc'[t], ii_hc'[t]} == {μ - (ma + μ) uu_n[t] -
        (β δ ((1 - κ) (ii_hc[t] + ii_hm[t] + ii_hn[t]) + (ii_rn[t] + ii_rc[t] + ii_rm[t])) +
            β (κ (ii_hc[t] + ii_hm[t] + ii_hn[t]) + ii_wc[t] + ii_wm[t] + ii_wn[t])) uu_n[t],
      - (ma + θ + μ + ω + β δ ((1 - κ) (ii_hc[t] + ii_hm[t] + ii_hn[t]) +
                  (ii_rn[t] + ii_rc[t] + ii_rm[t]))) ii_wn[t] +
        β (κ (ii_hc[t] + ii_hm[t] + ii_hn[t]) + ii_wc[t] + ii_wm[t] + ii_wn[t]) uu_n[t], - ii_rn[t]
         (ma + θ + μ + β (κ (ii_hc[t] + ii_hm[t] + ii_hn[t]) + ii_wc[t] + ii_wm[t] + ii_wn[t])) +
        β δ ((1 - κ) (ii_hc[t] + ii_hm[t] + ii_hn[t]) + (ii_rn[t] + ii_rc[t] + ii_rm[t])) uu_n[t],
      - (ma + θ + μ) ii_hn[t] + ω ii_wn[t] +
        β δ ((1 - κ) (ii_hc[t] + ii_hm[t] + ii_hn[t]) + (ii_rn[t] + ii_rc[t] + ii_rm[t])) ii_wn[t] +
        β ii_rn[t] (κ (ii_hc[t] + ii_hm[t] + ii_hn[t]) + ii_wc[t] + ii_wm[t] + ii_wn[t]), - μ uu_m[t] -
        (β δ ((1 - κ) (ii_hc[t] + ii_hm[t] + ii_hn[t]) + (ii_rn[t] + ii_rc[t] + ii_rm[t])) +
            β (κ (ii_hc[t] + ii_hm[t] + ii_hn[t]) + ii_wc[t] + ii_wm[t] + ii_wn[t])) uu_m[t] +
        ma uu_n[t], - (θ + μ + ω + β δ ((1 - κ) (ii_hc[t] + ii_hm[t] + ii_hn[t]) +
                  (ii_rn[t] + ii_rc[t] + ii_rm[t]))) ii_wm[t] + ma (1 - η) ii_wn[t] +
        β (κ (ii_hc[t] + ii_hm[t] + ii_hn[t]) + ii_wc[t] + ii_wm[t] + ii_wn[t]) uu_m[t], ma ii_rn[t] -
        ii_rm[t] (θ + μ + β (κ (ii_hc[t] + ii_hm[t] + ii_hn[t]) + ii_wc[t] + ii_wm[t] + ii_wn[t])) +
        β δ ((1 - κ) (ii_hc[t] + ii_hm[t] + ii_hn[t]) + (ii_rn[t] + ii_rc[t] + ii_rm[t])) uu_m[t],
      - (θ + μ) ii_hm[t] + ma ii_hn[t] + ω ii_wm[t] +
        β δ ((1 - κ) (ii_hc[t] + ii_hm[t] + ii_hn[t]) + (ii_rn[t] + ii_rc[t] + ii_rm[t])) ii_wm[t] + ma
         η ii_wn[t] + β ii_rm[t] (κ (ii_hc[t] + ii_hm[t] + ii_hn[t]) + ii_wc[t] + ii_wm[t] + ii_wn[t]),
      θ ρ_u (ii_wc[t] + ii_wm[t] + ii_wn[t]) +
        (- μ - β δ ((1 - κ) (ii_hc[t] + ii_hm[t] + ii_hn[t]) + (ii_rn[t] + ii_rc[t] + ii_rm[t])) -
            β (κ (ii_hc[t] + ii_hm[t] + ii_hn[t]) + ii_wc[t] + ii_wm[t] + ii_wn[t])) uu_c[t],
      - (ma η + θ + μ + ω + β δ ((1 - κ) (ii_hc[t] + ii_hm[t] + ii_hn[t]) +
                  (ii_rn[t] + ii_rc[t] + ii_rm[t]))) ii_wc[t] + θ ρ_w (ii_wc[t] + ii_wm[t] + ii_wn[t]) +
        β (κ (ii_hc[t] + ii_hm[t] + ii_hn[t]) + ii_wc[t] + ii_wm[t] + ii_wn[t]) uu_c[t],
      θ (ρ_r + ρ_u) (ii_hc[t] + ii_hm[t] + ii_hn[t]) + θ (ii_rc[t] + ii_rm[t] + ii_rn[t]) +
        θ ρ_r (ii_wc[t] + ii_wm[t] + ii_wn[t]) -
        ii_rc[t] (θ + μ + β (κ (ii_hc[t] + ii_hm[t] + ii_hn[t]) + ii_wc[t] + ii_wm[t] + ii_wn[t])) +
        β δ ((1 - κ) (ii_hc[t] + ii_hm[t] + ii_hn[t]) + (ii_rn[t] + ii_rc[t] + ii_rm[t])) uu_c[t],
      - (θ + μ) ii_hc[t] + θ (ρ_h + ρ_w) (ii_hc[t] + ii_hm[t] + ii_hn[t]) + (ma η + ω) ii_wc[t] +
        β δ ((1 - κ) (ii_hc[t] + ii_hm[t] + ii_hn[t]) + (ii_rn[t] + ii_rc[t] + ii_rm[t])) ii_wc[t] +
        θ ρ_h (ii_wc[t] + ii_wm[t] + ii_wn[t]) +
        β ii_rc[t] (κ (ii_hc[t] + ii_hm[t] + ii_hn[t]) + ii_wc[t] + ii_wm[t] + ii_wn[t])},
    {uu_n[0], ii_wn[0], ii_rn[0], ii_hn[0], uu_m[0], ii_wm[0], ii_rm[0], ii_hm[0],
      uu_c[0], ii_wc[0], ii_rc[0], ii_hc[0]} == SystemInitial},
   {uu_n, ii_wn, ii_rn, ii_hn, uu_m, ii_wm, ii_rm, ii_hm, uu_c, ii_wc, ii_rc,
    ii_hc}, {t, 0, 1000}];
```

```
κ = 0.7;
{ma, θ, ω, μ, ρ_h, ρ_u, ρ_r, ρ_w, η, β, δ, κ} =
```

```
{ma, θ, ω, μ, ρ_h, ρ_u, ρ_r, ρ_w, η, βhκ, δhκ, κ} /. output /. ma -> 0.00889;
SystemInitial = vars /. output;
HighKappa = NDSolve[{{uu_n'[t], ii_wn'[t], ii_rn'[t], ii_hn'[t], uu_m'[t], ii_wm'[t], ii_rm'[t],
      ii_hm'[t], uu_c'[t], ii_wc'[t], ii_rc'[t], ii_hc'[t]} == {μ - (ma + μ) uu_n[t] -
       (β δ ((1 - κ) (ii_hc[t] + ii_hm[t] + ii_hn[t]) + (ii_rn[t] + ii_rc[t] + ii_rm[t])) +
          β (κ (ii_hc[t] + ii_hm[t] + ii_hn[t]) + ii_wc[t] + ii_wm[t] + ii_wn[t])) uu_n[t],
      - (ma + θ + μ + ω + β δ ((1 - κ) (ii_hc[t] + ii_hm[t] + ii_hn[t]) + (ii_rn[t] + ii_rc[t] + ii_rm[
                    t]))) ii_wn[t] + β (κ (ii_hc[t] + ii_hm[t] + ii_hn[t]) + ii_wc[t] + ii_wm[t] +
          ii_wn[t]) uu_n[t], - ii_rn[t] (ma + θ + μ + β (κ (ii_hc[t] + ii_hm[t] + ii_hn[t]) +
              ii_wc[t] + ii_wm[t] + ii_wn[t])) + β δ ((1 - κ) (ii_hc[t] + ii_hm[t] + ii_hn[t]) +
          (ii_rn[t] + ii_rc[t] + ii_rm[t])) uu_n[t], - (ma + θ + μ) ii_hn[t] + ω ii_wn[t] +
       β δ ((1 - κ) (ii_hc[t] + ii_hm[t] + ii_hn[t]) + (ii_rn[t] + ii_rc[t] + ii_rm[t])) ii_wn[t] +
       β ii_rn[t] (κ (ii_hc[t] + ii_hm[t] + ii_hn[t]) + ii_wc[t] + ii_wm[t] + ii_wn[t]), - μ uu_m[t] -
       (β δ ((1 - κ) (ii_hc[t] + ii_hm[t] + ii_hn[t]) + (ii_rn[t] + ii_rc[t] + ii_rm[t])) +
          β (κ (ii_hc[t] + ii_hm[t] + ii_hn[t]) + ii_wc[t] + ii_wm[t] + ii_wn[t])) uu_m[t] +
       ma uu_n[t], - (θ + μ + ω + β δ ((1 - κ) (ii_hc[t] + ii_hm[t] + ii_hn[t]) +
                (ii_rn[t] + ii_rc[t] + ii_rm[t]))) ii_wm[t] + ma (1 - η) ii_wn[t] +
       β (κ (ii_hc[t] + ii_hm[t] + ii_hn[t]) + ii_wc[t] + ii_wm[t] + ii_wn[t]) uu_m[t], ma ii_rn[t] -
       ii_rm[t] (θ + μ + β (κ (ii_hc[t] + ii_hm[t] + ii_hn[t]) + ii_wc[t] + ii_wm[t] + ii_wn[t])) +
       β δ ((1 - κ) (ii_hc[t] + ii_hm[t] + ii_hn[t]) + (ii_rn[t] + ii_rc[t] + ii_rm[t])) uu_m[t],
      - (θ + μ) ii_hm[t] + ma ii_hn[t] + ω ii_wm[t] +
       β δ ((1 - κ) (ii_hc[t] + ii_hm[t] + ii_hn[t]) + (ii_rn[t] + ii_rc[t] + ii_rm[t])) ii_wm[t] + ma
        η ii_wn[t] + β ii_rm[t] (κ (ii_hc[t] + ii_hm[t] + ii_hn[t]) + ii_wc[t] + ii_wm[t] + ii_wn[t]),
      θ ρ_u (ii_wc[t] + ii_wm[t] + ii_wn[t]) +
       (- μ - β δ ((1 - κ) (ii_hc[t] + ii_hm[t] + ii_hn[t]) + (ii_rn[t] + ii_rc[t] + ii_rm[t])) -
          β (κ (ii_hc[t] + ii_hm[t] + ii_hn[t]) + ii_wc[t] + ii_wm[t] + ii_wn[t])) uu_c[t],
      - (ma η + θ + μ + ω + β δ ((1 - κ) (ii_hc[t] + ii_hm[t] + ii_hn[t]) +
                (ii_rn[t] + ii_rc[t] + ii_rm[t]))) ii_wc[t] + θ ρ_w (ii_wc[t] + ii_wm[t] + ii_wn[t]) +
       β (κ (ii_hc[t] + ii_hm[t] + ii_hn[t]) + ii_wc[t] + ii_wm[t] + ii_wn[t]) uu_c[t],
      θ (ρ_r + ρ_u) (ii_hc[t] + ii_hm[t] + ii_hn[t]) + θ (ii_rc[t] + ii_rm[t] + ii_rn[t]) +
       θ ρ_r (ii_wc[t] + ii_wm[t] + ii_wn[t]) -
       ii_rc[t] (θ + μ + β (κ (ii_hc[t] + ii_hm[t] + ii_hn[t]) + ii_wc[t] + ii_wm[t] + ii_wn[t])) +
       β δ ((1 - κ) (ii_hc[t] + ii_hm[t] + ii_hn[t]) + (ii_rn[t] + ii_rc[t] + ii_rm[t])) uu_c[t],
      - (θ + μ) ii_hc[t] + θ (ρ_h + ρ_w) (ii_hc[t] + ii_hm[t] + ii_hn[t]) + (ma η + ω) ii_wc[t] +
       β δ ((1 - κ) (ii_hc[t] + ii_hm[t] + ii_hn[t]) + (ii_rn[t] + ii_rc[t] + ii_rm[t])) ii_wc[t] +
       θ ρ_h (ii_wc[t] + ii_wm[t] + ii_wn[t]) +
       β ii_rc[t] (κ (ii_hc[t] + ii_hm[t] + ii_hn[t]) + ii_wc[t] + ii_wm[t] + ii_wn[t])},
   {uu_n[0], ii_wn[0], ii_rn[0], ii_hn[0], uu_m[0], ii_wm[0], ii_rm[0], ii_hm[0],
     uu_c[0], ii_wc[0], ii_rc[0], ii_hc[0]} == SystemInitial},
  {uu_n, ii_wn, ii_rn, ii_hn, uu_m, ii_wm, ii_rm, ii_hm, uu_c, ii_wc, ii_rc, ii_hc}, {t, 0, 1000}];
```

```
kappaplot = Plot[{Evaluate[(ii_rn[t] + ii_rm[t] + ii_rc[t] + ii_hn[t] + ii_hm[t] + ii_hc[t]) /
      (1 - uu_n[t] - uu_m[t] - uu_c[t]) /. HighKappa],
    Evaluate[((ii_rn[t] + ii_rm[t] + ii_rc[t] + ii_hn[t] + ii_hm[t] + ii_hc[t]) /
       (1 - uu_n[t] - uu_m[t] - uu_c[t])) /. Model],
    Evaluate[(ii_rn[t] + ii_rm[t] + ii_rc[t] + ii_hn[t] + ii_hm[t] + ii_hc[t]) /
      (1 - uu_n[t] - uu_m[t] - uu_c[t]) /. LowKappa]},
   {t, 0, 30}, PlotRange → {0.16, 0.202}, PlotStyle →
    {{ColorData["Crayola"]["ForestGreen"], Thickness[0.0075]},
     {ColorData["Crayola"]["Shamrock"], Thickness[0.0075]},
     {ColorData["Crayola"]["ScreaminGreen"], Thickness[0.0075]}},
   PlotLegends → {"κ=0.3", "κ=0.5 (baseline)", "κ=0.7"},
   FrameLabel → {"time (years)", "rate of resistance"},
   PlotTheme → "Scientific",
   LabelStyle → Directive[FontFamily → "Times", Black, FontSize → 14]]
```

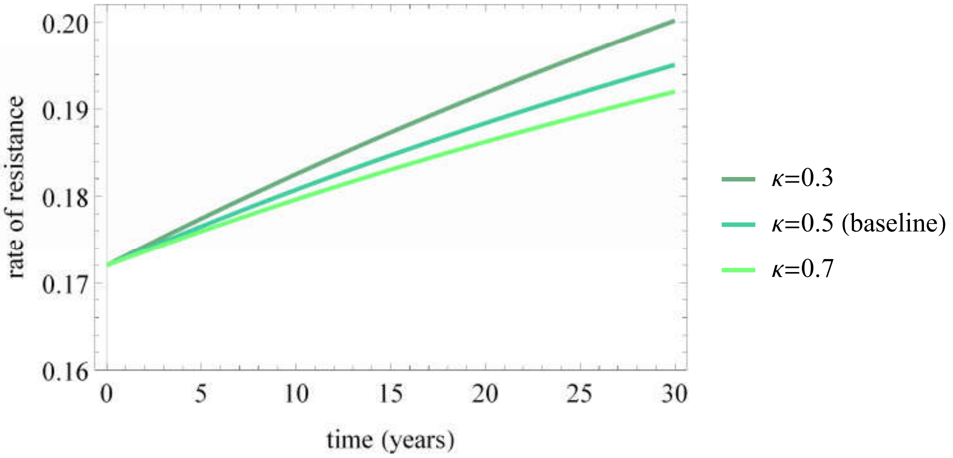


```
(*SetDirectory[NotebookDirectory[]];
Export["kappas.pdf",kappaplot,"pdf"]*)
```

Supplementary Note 4. Calculation of the number of newly evolving clarithromycin resistant (Cla-res) mutant bacteria in chronic *Helicobacter pylori* gastritis.

To estimate the number of newly emerging *Helicobacter pylori* (*H. pylori*) bacteria harbouring spontaneously evolved (non-Cla-treatment-related) Cla-res mutations, we first aimed to assess the bacterial load in chronic gastritis. For this purpose, it is necessary to know the size of the infected inner surface area of the stomach and the mucosal *H. pylori* concentration. Henry *et al* examined gastric volume and gastric surface area (GSA) data deriving from a series of computed tomography (CT) examinations of human subjects,[9] obtaining 154±65 cm^2 and 191±6 cm^2 GSA from patients with low and high body mass indices, respectively. However, CT does not detect fine details of mucosa; consequently, this approach does not take into consideration that rugae significantly increase the inner surface of stomach.

In order to obtain more precise GSA data, we measured the flattened-out stomach in ten regular autopsy cases in which a gross appearance of chronic gastritis had been found. The total mucosal surface area ranged between 55,900 mm^2 and 89,100 mm^2. Assuming that *H. pylori* colonizes between 20% and 70% of the total GSA (*H. pylori* is distributed in an uneven and patchy manner on the mucosa in chronic gastritis),[10,11] the infected area measures between 11,180 mm^2 and 62,370 mm^2. Based on Blaser and Kirschner's data, *H. pylori* concentration may range from 10^5 to 10^8 per mm^2 (assuming that the depth of bacterial invasion is 1 mm).[12] Thus, each stomach is estimated to contain between 1.118×10^9 and 6.237×10^{12} *H. pylori* bacteria.

The long-term mutation rate of *H. pylori* in the chronic phase of the infection has been reported to be 2.6×10^{-7} changes per site per year by Morelli *et al* and $0.5–2.5\times10^{-5}$ by Linz and colleagues.[4,13]. As a single nucleotide can be replaced by one of three other nucleotides, the chance of appearance of a specific mutation is one-third on the given site. On the other hand, the applied FISH method detects three distinct point mutations of the 23S rRNA gene, increasing this chance threefold. These two antagonistic effects neutralise each other: the rate of Cla-res mutations observable using our susceptibility test is equal to the general mutation rate. Accordingly, the number of newly emerging bacteria with a spontaneous Cla-res mutation can be calculated by multiplication of estimated total number of *H. pylori* (1.118×10^9–6.237×10^{12}) with the general mutation rate (2.6×10^{-7}–2.5×10^{-5} changes per site per year), giving a number between 290.68 and 1.56×10^8, meaning that at least 290 Cla-res bacteria appear in a stomach with chronic *H. pylori* gastritis per year. Even if we use the GSA data

published by Henry and colleagues,[9] at least 80 resistant bacteria emerge per year. At the other end of the scale, the number of Cla-res *H. pylori* bacteria evolving by spontaneous mutation might reach an order of magnitude of 10^6–10^8 per year.

Limitations of these results include the assumptions required for these calculations. Moreover, our FISH test was originally designed to detect the most prevalent point mutations found in clinical isolates[14] and we have considered only this three-nucleotide change even though further Cla-res-associated point mutations were identified in the 23S rRNA[15], as well as there being other, less common, mechanisms (deletions, RNA methylations, efflux pump, etc.) that can also lead to clarithromycin resistance.[16–19]

Accordingly, the number of newly evolving Cla-res mutant bacteria might be three times or more higher than results of the above calculation. Nevertheless, the final conclusion is clear: Cla-res *H. pylori* bacteria are continuously evolving by spontaneous mutation, even in the absence of selection pressure from macrolide antibiotics. Depending on the bacterial load and the actual mutation rate, at least hundreds and even up to 10^6–10^8 resistant bacteria will appear every year in a chronic gastritis case.

Probe name	Purpose of use	Targeted region	Detected mutation	Probe sequence
Hpy-1	*H. pylori*-specific probe	16S rRNA	–	5′-CACACCTGACTGACTATCCCG-3′
ClaR1	Cla-resistance-specific probe	23S rRNA	A2143G * (A2142G)	5′-CGGGGTCTTCCCGTCTT-3′
ClaR2	Cla-resistance-specific probe	23S rRNA	A2144G * (A2143G)	5′-CGGGGTCTCTCCGTCTT-3′
ClaR3	Cla-resistance-specific probe	23S rRNA	A2143C * (A2142C)	5′-CGGGGTCTTGCCGTCTT-3′
ClaWT	Cla-suceptibility-specific probe (wild type)	23S rRNA	–	5′-CGGGGTCTTTCCGTCTT-3′

Supplementary Table 8

List of the oligonucleotide probes used for the *Helicobacter pylori* (*H. pylori*) clarithromycin (Cla) susceptibility fluorescence *in situ* hybridization (FISH) test.

* The position of these two mutation sites were originally described as 2058 and 2059, based on the *Escherichia coli* 23S rRNA sequence. Subsequently, these were changed to 2143 and 2144 positions of the *H. pylori* 23S rRNA sequence. After a revision of this, the 2142 and 2143 numbering is mainly used currently.[20]

Supplementary References

1. Kocsmár, É. *et al.* Helicobacter pylori heteroresistance to clarithromycin in adults-New data by in situ detection and improved concept. *Helicobacter* **25**, e12670 (2020).
2. Chen, Q. *et al.* Randomised controlled trial: susceptibility-guided therapy versus empiric bismuth quadruple therapy for first-line Helicobacter pylori treatment. *Aliment. Pharmacol. Ther.* **49**, 1385–1394 (2019).
3. Yu, L. *et al.* Susceptibility-guided therapy for Helicobacter pylori infection treatment failures. *Therap. Adv. Gastroenterol.* **12**, (2019).
4. Linz, B. *et al.* A mutation burst during the acute phase of Helicobacter pylori infection in humans and rhesus macaques. *Nat. Commun.* **5**, 4165 (2014).
5. Bálint, L. *et al.* Epidemiologic characteristics of Helicobacter pylori infection in southeast Hungary. *World J. Gastroenterol.* **25**, 6365–6372 (2019).
6. Buzás, G. M., Lotz, G., Schneider, F. & Józan, J. [Changing prevalence of Helicobacter pylori infection in the 9th district of Budapest. A retrospective endoscopic study, 1997-2012]. *Orv. Hetil.* **154**, 900–907 (2013).
7. National Statistical Office (Hungary) KSH mortality reports https://www.ksh.hu/docs/hun/xstadat/xstadat_eves/i_wnh001.html https://www.ksh.hu/docs/eng/xstadat/xstadat_annual/i_wdsd006a.html https://www.ksh.hu/docs/eng/xstadat/xstadat_annual/i_wdsd006b.html
8. Hooi, J. K. Y. *et al.* Global Prevalence of Helicobacter pylori Infection: Systematic Review and Meta-Analysis. *Gastroenterology* **153**, 420–429 (2017).
9. Henry, J. A., O'Sullivan, G. & Pandit, A. S. Using computed tomography scans to develop an ex-vivo gastric model. *World J. Gastroenterol.* **13**, 1372–1377 (2007).
10. Engstrand, L. *et al.* Topographic mapping of Helicobacter pylori colonization in long-term-infected pigs. *Infect. Immun.* **60**, 653–656 (1992).
11. Misra, V. *et al.* A topographic study of Helicobacter pylori density, distribution and associated gastritis. *J. Gastroenterol. Hepatol.* **15**, 737–743 (2000).
12. Blaser, M. J. & Kirschner, D. Dynamics of Helicobacter pylori colonization in relation to the host response. *Proc. Natl. Acad. Sci. U S A* **96**, 8359–8364 (1999).
13. Morelli, G. *et al.* Microevolution of Helicobacter pylori during prolonged infection of single hosts and within families. *PLoS Genet.* **6**, e1001036 (2010).
14. Trebesius, K. *et al.* Rapid and specific detection of Helicobacter pylori macrolide resistance in gastric tissue by fluorescent in situ hybridisation. *Gut* **46**, 608–614 (2000).
15. De Francesco, V. *et al.* Change of point mutations in Helicobacter pylori rRNA associated with clarithromycin resistance in Italy. *J. Med. Microbiol.* **63**, 453–457 (2014).
16. Hirata, K. *et al.* Contribution of efflux pumps to clarithromycin resistance in Helicobacter pylori. *J. Gastroenterol. Hepatol.* **25**, S75–S79 (2010).

17. Liu, M. & Douthwaite, S. Activity of the ketolide telithromycin is refractory to Erm monomethylation of bacterial rRNA. *Antimicrob. Agents Chemother.* **46**, 1629–1633 (2002).
18. Webber, M. A. & Piddock, L. J. V. The importance of efflux pumps in bacterial antibiotic resistance. *J. Antimicrob. Chemother.* **51**, 9–11 (2003).
19. Mamelli, L., Prouzet-Mauléon, V., Pagès, J.-M., Mégraud, F. & Bolla, J.-M. Molecular basis of macrolide resistance in Campylobacter: role of efflux pumps and target mutations. *J. Antimicrob. Chemother.* **56**, 491–497 (2005).
20. Mégraud, F. Epidemiology and mechanism of antibiotic resistance in Helicobacter pylori. *Gastroenterology* **115**, 1278–1282 (1998).
