# Supplementary material for: Primary and secondary clarithromycin resistance in Helicobacter pylori and mathematical modeling of the role of macrolides
Source: Nat Commun. 2021 Apr 15;12:2255. doi: 10.1038/s41467-021-22557-7 (PMC8050269; doi:10.1038/s41467-021-22557-7)
Supplement: Supplementary file 3 — Description of Additional Supplementary Files [file 41467_2021_22557_MOESM3_ESM.pdf]

Description of Additional Supplementary Files

File Name: Supplementary Software 1
Description: The software code in a .zip file in .nb file format together with a readme.txt file with instructions for installing and running it in Wolfram Mathematica software (Supplementary Software 1).
